# Linking Plant Secondary Metabolites and Plant Microbiomes: A Review

**DOI:** 10.3389/fpls.2021.621276

**Published:** 2021-03-02

**Authors:** Zhiqiang Pang, Jia Chen, Tuhong Wang, Chunsheng Gao, Zhimin Li, Litao Guo, Jianping Xu, Yi Cheng

**Affiliations:** ^1^Institute of Bast Fiber Crops and Center of Southern Economic Crops, Chinese Academy of Agricultural Sciences, Changsha, China; ^2^CAS Key Laboratory of Tropical Plant Resources and Sustainable Use, Xishuangbanna Tropical Botanical Garden, Chinese Academy of Sciences, Kunming, China; ^3^College of Life Sciences, University of Chinese Academy of Sciences, Beijing, China; ^4^Department of Biology, McMaster University, Hamilton, ON, Canada

**Keywords:** root exudates, SynCom, multi-omics, phytohormones, VOCs, rhizobia, endophytes, pathogens

## Abstract

Plant secondary metabolites (PSMs) play many roles including defense against pathogens, pests, and herbivores; response to environmental stresses, and mediating organismal interactions. Similarly, plant microbiomes participate in many of the above-mentioned processes directly or indirectly by regulating plant metabolism. Studies have shown that plants can influence their microbiome by secreting various metabolites and, in turn, the microbiome may also impact the metabolome of the host plant. However, not much is known about the communications between the interacting partners to impact their phenotypic changes. In this article, we review the patterns and potential underlying mechanisms of interactions between PSMs and plant microbiomes. We describe the recent developments in analytical approaches and methods in this field. The applications of these new methods and approaches have increased our understanding of the relationships between PSMs and plant microbiomes. Though the current studies have primarily focused on model organisms, the methods and results obtained so far should help future studies of agriculturally important plants and facilitate the development of methods to manipulate PSMs–microbiome interactions with predictive outcomes for sustainable crop productions.

## Introduction

### Plant Secondary Metabolites

Different from primary metabolism, secondary metabolism refers to metabolic pathways and their associated small molecular products that are non-essential for the growth and reproduction of the organism (Yang et al., 2018). In plants, the secondary metabolic pathways produce a diversity of compounds called plant secondary metabolites (PSMs). PSMs contain a large group of structurally diverse compounds originated from either primary metabolites or intermediates in the biosynthetic pathways of these primary metabolites (Piasecka et al., 2015). According to their biosynthetic pathways, PSMs are generally classified into several large molecular families: phenolics, terpenes, steroids, alkaloids, and flavanoids (Kessler and Kalske, 2018).

Plant secondary metabolites play a variety of functions such as in plant growth and developmental processes, innate immunity (Piasecka et al., 2015), defense response signaling (Isah, 2019), and response to environmental stresses (Yang et al., 2018). In addition, PSMs also have important functions such as repelling pests and pathogens, acting as signals for symbiosis between plants and microbes, and modifying microbial communities associated with hosts (Guerrieri et al., 2019). Many PSMs have positive beneficial effects on human health (Ullrich et al., 2019; Fakhri et al., 2020) and on agriculture production, contributing significantly to the economy. However, the functions of many PSMs remain unknown. For example, while many PSMs and protein-metabolite complexes have been identified, the biological roles of most have not been verified (Kosmacz et al., 2020). There have been several excellent reviews summarizing recent studies reporting the novel roles of PSMs and emphasizing the importance of functional understanding of the plant metabolome (Fang et al., 2019; Kosmacz et al., 2020; Zhou and Pichersky, 2020). The studies presented in those reviews have benefited significantly from recent developments in omics technologies such as high throughput DNA sequencing and high-resolution mass spectrometry.

### Technologies for Analyzing Plant Metabolites

Plant metabolomics methods have been used for identifying functional secondary metabolites and metabolic pathways for both basic and applied research. Those methods help provide comprehensive perspectives on how plant metabolic networks are regulated. The most widely used methods include gas chromatography (GC) -mass spectrometry (-MS) (GC-MS), liquid chromatography-MS (LC-MS), capillary electrophoresis-MS (CE-MS), nuclear magnetic resonance spectroscopy (NMR), Fourier transform-near-infrared (FT-NIR) spectroscopy, MS imaging (MSI), and live single-cell -MS (LSC-MS). These methods are often used in combination because they can provide largely complimentary information with each other by analyzing different types of metabolites. A number of excellent technical reviews (Lu et al., 2017; Tahir et al., 2019) and detailed protocols (Zhalnina et al., 2018) regarding the utilization of these analytical tools in metabolomics experiments have been published.

Most traditional studies of PSMs utilize extracts of representative plant tissues as the main materials representing average plant cells in a specific tissue or organ (Masuda et al., 2018). Because of the bulk nature of those samples, it is often difficult to distinguish between PSMs produced by either host plants or their associated microbes. However, at present, there is a growing interest in narrowing PSMs analyses down to the single-cell level, allowing the separation of plant cells from their potentially associated microbial cells. Such separations and individualized analyses can be achieved using approaches such as MSI (Boughton and Thinagaran, 2018), matrix-assisted laser desorption ionization (MALDI) and laser ablation electrospray ionization (LAESI) (Etalo et al., 2018a; Bhattacharjee et al., 2020), live single-cell mass spectrometry (LSC-MS) (Masuda et al., 2018), nanospray desorption electrospray ionization mass spectrometry (Nano-DESI MS) (Battin et al., 2016), and the spatial metabolomics pipeline (metaFISH) (Geier et al., 2020). In combination with MS data alignment and molecular networking software and relevant databases, these tools allow for the detection of a large number (hundreds to thousands) of metabolites acquired from a single plant cell (Brader et al., 2014). These platforms provide significant advancement for the discovery of metabolites produced *in situ* and of the dynamics of interactions between plant and microbial cells at a single-cell level.

### Plant Microbiome

The microbial communities of plants, also known as the plant microbiome (or plant microbiota), are found in the rhizosphere, phyllosphere, and endosphere. These plant microbiomes play important roles in helping host plants develop immunity (Stringlis et al., 2018), suppress diseases (Carrion et al., 2019), supply nutrients (Zhang et al., 2019), and protect from biotic and abiotic environmental stresses (de Vries et al., 2020). Over the last 15 years, plant microbiome studies have progressed significantly due to the advent of massive parallel sequencing. These studies have helped define different kinds of plant microbiomes and plant–microbiome interactions, e.g., the epiphytic microbiome, seed microbiome, core microbiome (CM), synthetic community (SynCom), and DefenseBiome (Liu H. et al., 2020). However, these plant microbiomes are not static, they can change in response to environmental stimuli, including both abiotic stresses and biotic factors. Indeed, there is increasing evidence that the structure of plant microbiomes is the result of a series of forward and backward interactions between the plant, the microbes and their environmental physical and chemical conditions. For example, PSMs secreted by roots are important mediators of plant–soil microbiome interactions (Sasse et al., 2018). In maize, secondary metabolites such as benzoxazinoids, were shown to attract bacteria *Chloroflexi* and influence the assembly of the maize microbiomes that subsequently enhance the capacity of maize plants to adapt to their environments (Hu et al., 2018).

Among the plants analyzed so far, model species such as *Arabidopsis thaliana* and *Echinacea purpurea* have been extensively studied to help define their microbiomes and the roles of these microbiomes in enhancing the growth and reproduction of host plants (Kudjordjie et al., 2019; Maggini et al., 2020). However, despite the growing number of studies and reviews demonstrating that different host plants species (Compant et al., 2019), their development stages (Schlechter et al., 2019), their root exudates (Sasse et al., 2018; Vives-Peris et al., 2020), and their rhizodeposits (Tian et al., 2020) can all influence the composition of the plant microbiomes and their functional capacities, relatively few reviews have attempted to integrate the chemical basis and molecular mechanism into the PSMs-microbiome relationship. Complicating the understanding is that the relationship is a dynamic one, involving multiple back-and-forth exchanges of chemical signals and molecular pathways. For example, some microbes can modulate the production of PSMs, including plant bioactive phytometabolites that in turn can influence the microbiome (Mastan et al., 2019). Furthermore, while a number of studies have provided insights into the structure and dynamics of the plant microbiome, relatively little is known about the contribution of plant microbiomes to host PSMs. The latest models of traditional medicinal PSMs–microbiome interactions approaches provide a new framework for understanding the various types of interactions between PSMs and microbiomes (Maggini et al., 2020). Such an understanding can have significant impacts on several applied fields such as crop cultivation and breeding. During crop breeding, scientists typically select for higher yield and/or better nutrition but only from the crop cultivar perspective with limited consideration of the plant microbiome or the PSMs–microbiome interactions. Understanding of the interactions between PSMs and plant microbiomes could help opening up a new avenue of research in crop production.

Over the past few years, the characterization of the plant microbiomes and their relationships with the host plants using high-throughput techniques including genome and metagenome sequencing has become a hot topic in research. Both the culturome (all microbes that can be cultured in the lab) and metagenome sequencing techniques are providing in-depth information of the plant microbiome. The culturome is an important component of the microbiome. To obtain the culturome, the culturable cells in the microbial community are selected using solid media or liquid medium in high throughput formats. Subsequent shotgun sequencing allows the identification of linkages between taxonomic identity to important functions to the cultured cells, such as biological nitrogen fixation. However, culture-based methods are usually less sensitive than direct amplicon sequencing for identifying rare microbes. High-throughput sequencing of specific gene amplicons is typically more powerful for elucidating the composition, and spatial distribution of microorganisms in their environments and this approach is increasingly used in plant microbiome studies. The metagenome approaches can be combined with other high-throughput methods, such as metabolomics, proteomics, and transcriptomics. There is an excellent review discussing the quality of publicly available genome data, metagenome data, other omics data, and software pipelines for analyzing such data (Lucaciu et al., 2019). In these analyses, it’s important to minimize sequence artifacts and reduce noise in data (Davis et al., 2018; Zhou et al., 2019). For processing the bacterial 16S rRNA gene and fungal ITS amplicons, a collection of software, such as QIIME (Caporaso et al., 2010), UPARSE (Edgar, 2013), VSEARCH (Rognes et al., 2016), PIPITS (Gweon et al., 2015), and USEARCH (Edgar and Flyvbjerg, 2015) have been developed. Similarly, for shotgun microbiome sequencing analyses, several recent articles reported specific computational workflow and bioinformatics resources (Liu Y. X. et al., 2020), including Microbiome Helper (Comeau et al., 2017), HmmUFOtu (Zheng et al., 2018), iMicrobe (Youens-Clark et al., 2019), MMinte (Mendes-Soares et al., 2016), MDiNE (McGregor et al., 2020), MicrobiomeAnalyst (Dhariwal et al., 2017), SIMBA (Mariano et al., 2016), and iMAP (Buza et al., 2019). Several in-depth summaries and comparisons of next-generation amplicon sequencing and analyses approaches were published recently (Lucaciu et al., 2019; Nilsson et al., 2019).

Herein, we review the current literature on the bidirectional interactions and effects between PSMs and plant microbiomes. In addition, we review the latest advances in plant metabolome analytical technologies and methods for analyzing the relationships between the plant metabolome and the plant microbiomes. To achieve our objectives, we used the following keywords for database searches: a variety of plants (such as legume plants, medicinal plants), plant microbiome (microbiota), metagenome, metagenomic, amplicon sequencing, PSMs, metabolomics, metabolomic analytical methodology, plant metabolome databases, correlation relationship, metabolomic-microbiome, and omics etc. The main retrieval databases were Web of Science, PubMed, and ResearchGate. Table 1 shows a few common terms and their definitions used in this review.

**TABLE 1:** Common terms and definitions.

**Term**	**Definition**
Secondary metabolism	Metabolic pathways and their associated small molecular products that are non-essential for the growth and reproduction of the organism
Rhizosphere microbiome	All microorganisms found in the narrow region of soil or substrate that is directly influenced by root secretions and associated soil, also called root microbiome
Epiphytic microbiome	All microorganisms found on the surface of aerial parts of plants. These microorganisms use plants for physical support but do not obtain any nutrients from plants nor cause any damage or offer any benefit to host plants.
Endophytic microbiome	All microorganisms found inside the internal tissues of plants, including both aerial and root tissues
Seed microbiome	All microorganisms found on the surface of and inside the seeds
Core microbiome	The group of microorganisms that are found in all individuals of a host species. The persistent association suggests a potentially critical function within the ecological niche of the host in which these microorganisms are found
Synthetic community (SynCom)	Defined systems with reduced complexity for both the host and the microorganisms. SynCom serves as model systems to investigate the performance and stability of microbial communities or to identify the necessary conditions for generating interaction patterns and higher order community structure and function
DefenseBiome	Plant-associated microbes that are positively associated with plant stress resistance
Rhizodeposits	All material transferred from plant roots to the soil. They include dead root tissues and cells, root exudates (both soluble and insoluble materials), and gasses such as CO_2_ and ethylene.
Culturome	All microbes in a sample that can be cultured in the lab.

### Data Analyses Tools for Association Studies Between Plant Metabolome and Microbiome

Due to advances in high-throughput sequencing techniques, direct analyses of microbial communities in their natural environments have become increasingly convenient and cost effective. In recent years, microbiome studies using multi-omics approaches have greatly deepened our understanding of the relationship between microbiomes and hosts. For example, multi-omics studies of the gut microbiome and the human metabolome (Chen M. X. et al., 2019; Ilhan et al., 2020) have provided new understanding in human health and diseases. In order to help the application of multi-omics technologies on plant metabolome and microbiome studies, we reviewed the data integration and analysis methods for studying human and animal microbiomes and metabolomes; and provided a few suggestions on how they could be used for plant studies. Table 2 lists the methods and tools for association studies between metabolome and microbiome.

**TABLE 2:** Comparison of methods and tools for association studies between metabolites and microbiomes.

**Type of analysis**	**Method**	**Function**	**Note (Link)**	**References**
Univariate analysis	Pearson Spearman	Comparatively simple method, but high false positive rate, unable to explain biological mechanism	Multiple calibration tests are required	Mao et al., 2016; Ilhan et al., 2020
Common descending dimension methods	PCA PLS	A multivariable correlation analysis method to describe the relationship between the two data matrices.		van der Kloet et al., 2016
Joint and individual variation explained	JIVE	An extension of PCA, identifying joint variations in multiple data types, reducing the dimensionality of data and determining the unique features of a particular data type.		Lock et al., 2013
Simultaneous component analysis	SCA	DIStinct COmmon SCA (DISCO-SCA) offers new avenues for multi-omics data fusion		Smilde et al., 2017
Canonical correlation	CCA	Samples over variables, Sparse CCA, kernel CCA and RCCA	Multivariable analysis partial least-squares	Kostic et al., 2015
Procrustes analysis	PA	Powerful least-square approach, simplifies omics comparisons, may not be sufficient to draw conclusions		McHardy et al., 2013
Co-inertia analysis	CIA	Suitable approach to determine the relationship, not suitable for large-scale data analysis		Hill et al., 2017
Maximum information coefficient	MIC	MINE statistics for identify associations and characterize such as non-linearity and monotonicity, comes closer than mutual information.	http://exploredata.net	Reshef et al., 2011
Linear regression	LR	Provide more accurate results		Bakker O. B. et al., 2018
Generalized coRrelation analysis	GRaMM	Captures linear/non-linear correlations in datasets and can adjust the influence of confounders, combines LR, MIC et al.	https://github.com/chentianlu.	Liang et al., 2019
Seed set framework	A TDA	Calculate the symbiosis and competition scores of different microbes and predict the interaction relationship between microbes.		Greenblum et al., 2012
Predicted relative metabolic Turnover	PRMT	Explore metabolite-space inferred from the metagenome, can combine and analyze additional metagenomic and metabolic datasets	http://camera.calit2.net	Larsen et al., 2011
Computational framework	MIMOSA2	Mechanistic interpretation and hypothesis generation. Only analyze metabolites in the specific reference database	www.borensteinlab.com/software_MIMOSA2.html	Noecker et al., 2016
Genome-scale metabolic models	GEMs	Contains a complete metabolic map of all metabolic reactions of the body and can be used for metabolite. Integration of Histology and Metagenomics Data		Magnúsdóttir et al., 2017
Neural networks	Mmvec	Estimate probability and strength of interaction	https://github.com/biocore/mmvec)	Morton et al., 2019
A Valid Alternative to Correlation	Proportionality	Present proportionality as a means to analyze related data.		Lovell et al., 2015
Correlation inference for Compositional data through Lasso	CCLasso	An alternating direction algorithm from augmented Lagrangian method. Poorly for the hub model, component fraction estimation	https://github.com/huayingfang/CCLasso	Fang et al., 2015
Sparse Correlations for Compositional data	SparCC	Not rely on high diversity. Relies on reliable component counts, no considered for the overall property	https://bitbucket.org/yonatanf/sparcc	Friedman and Alm, 2012
SParse InversE Covariance Estimation	SPIEC-EASI	Making assumptions about the underlying network structure. Scale-free structures elude accurate inference	http://bonneaulab.bio.nyu.edu/	Kurtz et al., 2015
Correlation network	CCREPE	(bioBakery or ReBoot) Provides a similarity measure more appropriate for compositional data analysis, performance is similar to SparCC	http://huttenhower.sph.harvard.edu/ccrepe	Faust et al., 2012; McIver et al., 2018
Multivariate statistical analyses	M^2^IA	Integrative data analysis from data preprocessing, univariate and multivariate statistical analyses, advanced functional analysis for biological interpretation, to a summary report.	http://m2ia.met-bioinformatics.cn	Ni et al., 2020

**FIGURE 1:**
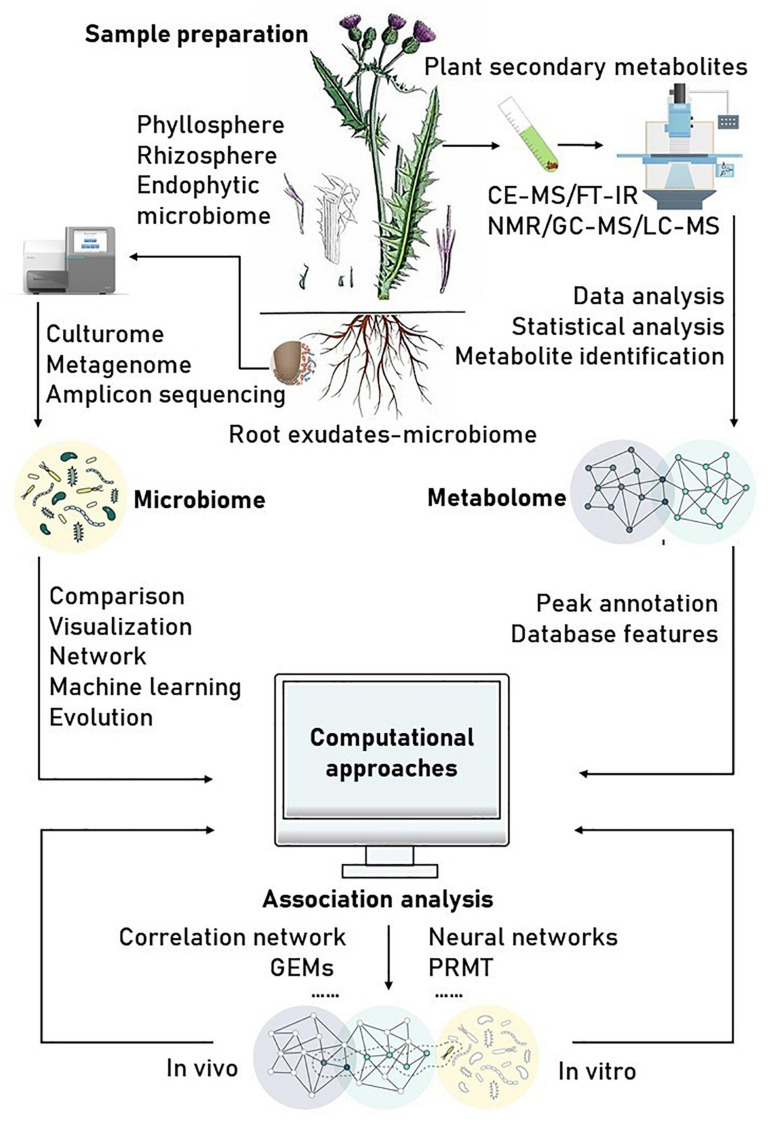
Reference of schematic workflow for plant secondary metabolomics-microbiome discovery projects. Some information and pictures are adapted from Lavelle and Sokol (2020).

Correlation-based analysis of paired microbiome-metabolite data sets has been a common approach to identify microbial drivers of metabolic variations. A commonly used method to infer the drivers of metabolic variations in a network is correlation analysis, such as Pearson’s and Spearman’s correlation coefficients among all pairs of operational taxonomic units (OTUs) and the metabolite profiles. An interaction between microbes is inferred when there is a high correlation coefficient between them (Morton et al., 2019). However, traditional correlation analyses, such as univariate analysis and simultaneous component analysis that treat the observed data as absolute abundances of the microbes, may lead to spurious results. This is because most of the observed data through metagenomic analyses only represent relative abundances (Gevers et al., 2014). For example, concluding that a microbial community showing no signs of microbiome–metabolite interactions based on a single correlation analysis is unlikely correct, as none of the traditional tools can definitively identify actual correlations (Weiss et al., 2016). Consequently, simple correlation analysis alone is not suitable for detecting true microbial contributors to metabolite variations. Thus, special care and appropriate adjustments are required prior to correlation analysis for microbiome and metabolome data (Fang et al., 2015). Recent methods such as MIMOSA2, Correlation inference for Compositional data through Lasso (CCLasso), Neural networks (such as mmvec), Predicted relative metabolic turnover (PRMT), Compositionally Corrected by REnormalization and PErmutation (CCREPE), and Sparse Correlations for Compositional data (SparCC) (Table 2) have been designed to take these compositional biases into account for analyzing microbiome–metabolite interactions. The joint usage of multiple methods can achieve better results. Several tools and resources are described in Figure 1 and the following subsection.

Parallel approaches can offer new opportunities for analyzing microbiome–metabolite interactions especially if diverse types of information can be integrated. However, such data integration and analysis methods are still in their early stages of development (Lamichhane et al., 2018). In addition, to understand the underlying biological processes for the observed patterns of interaction, it is important to develop computational approaches that include individual organisms’ unique biological features (Mallick et al., 2019). With a growing interest in connecting the microbes and metabolites in the context of plant and human health, we also need to bring together researchers from the two domains that traditionally do not interact with each other (Misra, 2020).

Importantly, while there are limitations in the correlation-based analysis to identify key microbiome-metabolite links, such linkages can be found in the current microbiome-metabolome data (Noecker et al., 2019). As is commonly stated, a correlation doesn’t mean a causation or a true biological interaction. However, statistically significant correlations do help generate hypotheses and guide experimental efforts. Indeed, appropriately designed and carefully executed experiments are indispensable for confirming the hypotheses about the role of specific metabolites in plant–microbiome interactions. In the sections below, we first describe evidence for and a general model of PSMs–plant microbiome interactions. We then use specific examples to show how PSMs influence plant microbiomes (see section “Evidence for Specific PSMs Modulating the Plant Microbiome”) and how plant microbiomes influence PSMs (see section “Plant Microbiomes Contribute to the Productions of PSMs”). We then describe how the PSMs–plant microbiome interactions could be used for crop production (see section “PSMs–Microbiome Interactions Impact Crop Breeding, Abiotic Stress Response, and Plant Invasion”). We finish by discussing potential areas for future research.

## Evidence and Model Framework for Interactions Between Plant Secondary Metabolites and Plant Microbiomes

**FIGURE 2:**
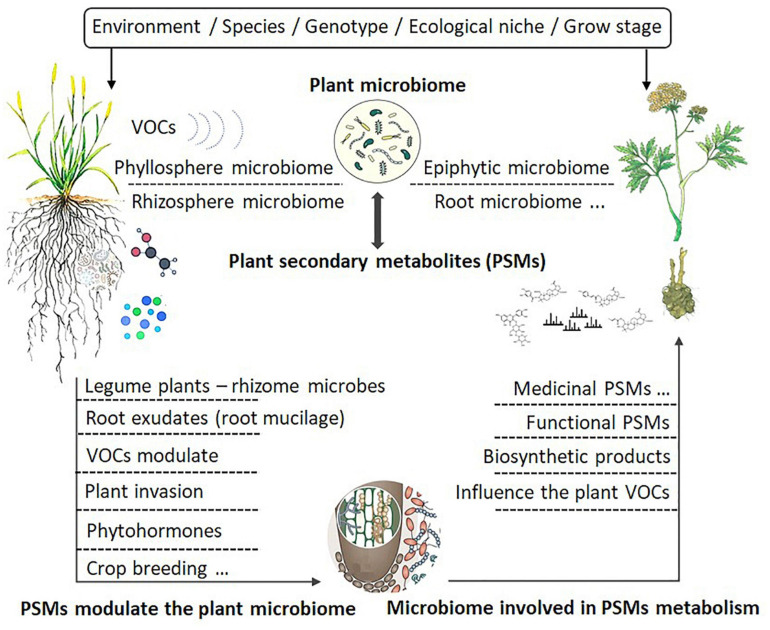
Factors influencing the interactions between plant secondary metabolites and plant microbiomes.

As shown by Köberl et al. (2013), the same plants grown in different locations may produce different SMs, with some of the differences attributed to their associated microbes at different sites. Microbes adapted to specific locations and associated with specific plants may produce unique effects on host plants, including the production of SMs (Huang et al., 2018). For example, *Methylobacterium* was found to be involved in modulating the production of phytometabolites associated with flavor and in metabolizing plant host compounds, including volatile organic compounds (VOC) (Brader et al., 2014). Indeed, the induction of PSMs by endophytes may be a very general phenomenon in aromatic and medicinal plants. For example, several studies have shown that root exudates containing compounds such as aromatic organic acids (nicotinic, shikimic, salicylic, cinnamic, and indole-3-acetic acids) could shape the root microbiome (rhizobiome), which subsequently influenced root–microbe interactions (Sasse et al., 2018; Cotton et al., 2019). The combinations of plant exudation and microbial nutrient traits could interact to produce unique microbial community assemblies (Zhalnina et al., 2018). These studies have led to a proposed framework for studying the relationship between microbiome and PSMs, as depicted in Figure 2.

Interactions between legume plants and their rhizobia represent among the best studied models of PSMs-microbe interactions. Besides being economically important crops for food and forage, legume plants (such as pea, soybean, peanuts, clover, and alfalfa) and rhizobia have been used for decades for revealing how secondary metabolites from both partners mediate their interactions to establish root nodules for biological nitrogen fixation. Previous studies have observed a higher abundance of symbiotic rhizobia in the root microbiome of legume plants (70% with clover *Trifolium*) than that of bulk soil or the root microbiome of other plants (Hartman et al., 2017; Zhang et al., 2018). Soybean (*Glycine max*) is an example of legume plants that has been studied extensively for its mutualistic relationships with nitrogen-fixing rhizobia and arbuscular mycorrhizal fungi. Soybeans secrete various specialized metabolites such as isoflavones and saponins into the soil (Sugiyama, 2019). Specifically, isoflavones and strigolactones are signal molecules for symbioses between soybean with rhizobia and arbuscular mycorrhizal fungi, respectively. During symbiosis, a hallmark feature of legume plants is that their roots secrete flavonoids/isoflavones [such as condensed tannins (CTs, prodelphinidins and procyanidins), daidzein and genistein)] into the rhizosphere as signal compounds to attract nitrogen-fixing bacteria such as *Ensifer* (formerly *Sinorhizobium*), *Rhizobium*, *Allorhizobium*, *Mesorhizobium*, *Neorhizobium*, *Azorhizobium*, *Pararhizobium*, and *Bradyrhizobium* (Hartman et al., 2017). Similarly, bacteria in the genera *Cupriavidus*, *Paraburkholderia*, and *Trinickia* also form mutualistic interactions with Papilionoideae and Caesalpinioideae to establish nodulation. The analysis of rhizosphere microbiomes between plants with and without isoflavone synthetase revealed that isoflavones exerted significant influence on the abundance of Xanthomonadaceae and Comamonadaceae (White et al., 2017). In addition, a recent study indicated that daidzein had a significant effect on soybean root microbiome, showing a concentration-specific effect on the bacterial community assemblage (Okutani et al., 2020). Specifically, the results suggested that daidzein functions both as an attractant and a repellent for different groups of bacteria. When the concentration of daidzein is high in the rhizosphere, there is an increased abundance of Comamonadaceae while rhizobia abundance was decreased, causing an overall reduced α-diversity. The overall reduced microbial diversity was probably because daidzein is not a preferred carbon source of rhizobia, different from several other bacteria that were enriched in daidzein-treated soils. In addition, a study showed that root–root interactions between the broad bean (*Vicia faba*) and maize significantly increased both nodulation and symbiotic N_2_ fixation in intercropped *V. faba* (Li et al., 2016). However, while root exudates from maize promoted faba bean nodulation (flavonoids), root exudates from wheat and barley did not (Li et al., 2016). Recently, an interesting study suggested that cyanide production by cassava (*Manihot esculenta* Crantz) can trigger ethylene production in adjacent peanut (*Arachis hypogaea* L.) roots (Chen Y. et al., 2020), which subsequently changed the microbial composition and re-assembled the microbial co-occurrence network of peanut plants, causing an increased abundance of *Catenulispora* spp., an actinobacterium. However, the full details of this specific relationship between legume plants and rhizobia remain to be elucidated.

Apart from impacting legume and bacterial interactions, flavanones (such as strigolactones) can positively influence the growth of ectomycorrhizal fungi and increase the colonization of AM fungi. For example, flavanones can enhance the germination of spores of ectomycorrhizal fungi in genera *Pisolithus* and *Suillus* as well as stimulate the production of symbiotic effector protein in the mushroom *Laccaria bicolor* (Garcia et al., 2015; Pei et al., 2020). In contrast, the suppression of flavonoids and phenylpropanoid pathway secretion reduced the endophytes and ectomycorrhizal colonization of the maize and poplar roots, respectively (Mehmood et al., 2020). At present, the mechanisms of their interactions remain unclear.

## Evidence for Specific PSMs Modulating the Plant Microbiome

Plants exude both low-molecular-weight compounds (such as phenolics, amino acids, nucleotides, sugars, terpenoids, and lipids) and high-molecular-weight compounds (such as nucleic acids, polysaccharides, and proteins). The types of compounds and their relative abundances depend on the species of plants, their growth and developmental stages, and presence of stress (abiotic, biotic) factors (Korenblum et al., 2020). The key classes of PSMs are either non-volatile compounds or VOCs. Plant roots secrete PSMs into the rhizosphere and/or soil environment actively using ATP as the energy source and passively through diffusion. PSMs are also released when root tissues and cells are detached from roots. After entering into the rhizosphere and soil, most PSMs may be quickly utilized by soil microbes, but some can remain in the rhizosphere and mediate interactions among organisms (Sugiyama and Yazaki, 2014). The roles of root exudates in plant–microbe chemical interactions in the rhizosphere is increasingly recognized (Sasse et al., 2018; Yuan et al., 2018; Williams and de Vries, 2020). Furthermore, different rhizodeposits can influence the rhizosphere microbiome composition differently (Pascale et al., 2020). Some root PSMs can affect the assembly of the root microbiome even before microbes reach the root surface (Sasse et al., 2018). Recent studies showed that selected SMs including coumarin, triterpenes, flavonoid, benzoxazinoid, and phytohormones can impact the proliferation or suppression of specific microbes around the root of host plants (Holmer et al., 2017; Hu et al., 2018; Chen Q. et al., 2019; Voges et al., 2019; Chen Q.-L. et al., 2020). These results call for further investigations into how natural habitat variation, crop genetic variation, and plant introduction between locations can potentially affect the PSMs and the recruitment and assembly of plant microbiome.

### Coumarin, Benzoxazinoid, Terpenes, and Other Root-Exuded Molecules Modulate Root Microbiome

Plant secondary metabolites capable of changing plant microbiomes belong to diverse classes, including phenolics, benzoxazinoids, terpenes, and alkaloids (Cotton et al., 2019; Voges et al., 2019; Wang and Niu, 2019). Non-volatile compounds such as coumarins and flavonoids are produced by many plant species and are common in the rhizosphere. Coumarins are a family of plant-derived SMs produced via the phenylpropanoid pathway, and are involved in responses of dicotyledonous plants to iron deficiency (Stringlis et al., 2019). Recent studies suggested that coumarins, a sub-group of phenolic compounds, can influence the rhizosphere microbiome composition and exhibit differential toxicity against beneficial and pathogenic microorganisms (Lundberg and Teixeira, 2018; Voges et al., 2019). For example, a coumarin-deficient *Arabidopsis* mutant with beta-glucosidase gene *BGLU42* knocked out showed an increase in the relative abundance of Proteobacteria and a decrease of Firmicutes around its root (Stringlis et al., 2018, 2019). Further experiments showed that one specific coumarin compound called scopoletin inhibited the growth of soilborne pathogens whereas rhizobacteria were not affected. This was further confirmed by evidence showing that coumarins could shape the composition of a SynCom, where the abundance of *Pseudomonas* was significantly higher in coumarin-deficient *Arabidopsis f6’h1* mutants than in wild-type plants (Voges et al., 2019). A recent excellent review described coumarins as the “new kids on the block” in the chemical communications between plant roots and root microbiomes (Stassen et al., 2020).

Recent studies found that benzoxazinoids (BXs), SMs produced by several *Poaceae* species, and several downstream metabolites, could act as allelochemicals and natural pesticides on the root microbiome (Hu et al., 2018; Cotton et al., 2019; Kudjordjie et al., 2019; Schütz et al., 2019; Jacoby et al., 2020). Specifically, Hu et al. (2018) used a benzoxazinoids deficient maize mutant *bx1* and found that different bacterial and fungal communities were assembled in the roots of the mutants compared to wild-type maize. Another research used different maize BX mutant materials (BX knockout mutants, *bx1, bx2*, and *bx6*, and their near-isogenic W22-based controls T43 and a_1_-m_3_), and found similar results (Kudjordjie et al., 2019). Interestingly, such effects could be detected over several generations of the maize crop, suggesting that these molecules are likely key agents in plant–soil microbe feedback interactions (Jacoby et al., 2020). Overall, benzoxazinoids enriched Methylophilaceae bacteria while repressed Xanthomonadaceae (Cotton et al., 2019), likely due to their differential ability to use benzoxazolinones as carbon and energy sources (Schütz et al., 2019).

Similar to the benzoxazinoids, camalexin, an indolic compound, can also modulate the functionality of root microbiome (Koprivova et al., 2019). Loss of function of *CYP71A27*, a root-specific gene involved in the synthesis of camalexin, affected not only the soil microbiome but also led to the loss of plant growth-promoting effect by *Pseudomonas*. However, loss of the growth-promoting effect in the *CYP71A27* mutants could be complemented chemically by the addition of camalexin. Taken together, these results suggested camalexin’s beneficial effects on plants by mediating the interaction between plant roots and microbes (Koprivova et al., 2019).

Terpenoids are a major component of the root-specialized metabolites. They contribute to the assembly of *Arabidopsis*-specific root microbiome by regulating the growth of specific root bacteria (Wang and Niu, 2019). One group of terpenoids is the triterpenes, synthesized via the mevalonate pathway that can accumulate in plant tissues as triterpene glycosides (Pascale et al., 2020). Recent experiments investigated the effects of triterpene and sesterterpene biosynthesis on *Arabidopsis* root microbiome assembly. The results showed that the compositions of the root microbiome communities of the triterpene and sesterterpene biosynthesis mutants were significantly different from those of the wild-type plants (Chen Q. et al., 2019; Huang et al., 2019). The authors further investigated whether triterpenes regulated *Arabidopsis* root bacteria using purified triterpene compounds. Growth assays of selected microbial strains showed that purified triterpenes stimulated the proliferation of *Arenimonas* while inhibited the growth of *Arthrobacter* (Huang et al., 2019).

Some of the known PSMs have potent antibiotic activities. Plants secreting such compounds may create additional constraints on the groups of bacteria that can grow in the specific plant microbiome (Huang et al., 2018). For example, flavonoids have potent antimicrobial activity against a wide range of pathogenic microorganisms *in vitro* (Górniak et al., 2019). In addition, some PSMs such as flavonoids are not only associated with the regulation of symbiosis between plants and microbes (e.g., arbuscular mycorrhizal, ectomycorrhizal, rhizobial, and actinorhizal symbioses), but also as quorum-sensing (QS) inducers for communications among microbes. Different legumes produce unique flavonoids that bind to specific NodD proteins on the surface of rhizobia strains and species to regulate their symbiosis (Holmer et al., 2017). Application of 7, 4′-dihydroxyflavone, the most abundant flavonoid in the root exudate of *Medicago sativa*, to bulk soil caused significant changes of the relative richness of *Acidobacteria* (increased), *Gaiella*, *Nocardioidaceae*, and *Thermomonosporacea* (reduced). These microbes are known to interact with plant roots (Szoboszlay et al., 2016). Similarly, catecholic coumarins, benzoxazinoid, terpenes, jasmonate, indole glucosinolates, daidzein and others compounds also have antibacterial activity (Maggini et al., 2018; Rajniak et al., 2018; Dubey et al., 2020). Decades of research have demonstrated that a large number of secondary metabolites are involved in plant defense response to pathogens (Wang et al., 2020). Indeed, some of these PSMs have been used in antimicrobial scaffolds (Trda et al., 2019). There is increasing interests in mining bioactive compounds from economic crops such as garlic and ginger who are rich in allicin and curcumin etc. as natural antimicrobial compounds in healthy foods.

### Volatile Organic Compounds From Plants Modulate Plant Microbiome

Apart from soluble secondary metabolites mentioned above, plants also release various VOCs which constitute an estimated 1% of PSMs (Venturi and Keel, 2016). Due to their unique physico-chemical properties, VOCs can easily diffuse through gas- and water- filled pores in the soil and can, therefore, have a broad effective range in soil (Schulz-Bohm et al., 2018), including mediating interactions with surrounding soil microorganisms. Examples of major plant VOCs include aldehydes, terpenoids, phenylpropanoids, and common monoterpenes limonene, β-pinene, benzenoids, and β-caryophyllene. Many of these VOCs have antimicrobial properties and can strongly influence plant microbiomes, including that on the plant phyllosphere (Farré-Armengol et al., 2016). A recent study evaluated the antimicrobial and anti-quorum sensing (QS) properties of 29 common essential oil compounds from plants. Twenty-two of these 29 compounds were identified to have QS-inhibitory ability, while seven promoted the QS to a variable extent in populations of two bacteria *Chromobacterium violaceum* and *Pseudomonas aeruginosa* (Peter et al., 2019). These results suggested that QS-inhibitory compounds of natural plant origins could be used to formulate a new generation of antimicrobial agents. In addition, another recent study found that the attraction of certain bacteria with antifungal properties through soil toward roots could be stimulated by VOCs (e.g., propanal, γ-nonalactone, and dimethyl disulfide) produced by *Carex arenaria* roots, with the cell numbers of *Janthinobacterium*, *Collimonas*, and *Paenibacillus* increased by up to three times higher as compared to those in the control soil samples without *C. arenaria* (Schulz-Bohm et al., 2018). However, the soil microbes were not equally attracted by VOCs to colonize plant roots.

Due to their antimicrobial effects and their potential role as carbon sources, plant VOCs can play significant roles in determining the characteristics of the microbiome on the above-ground plant surfaces (including stems, leaves, flowers, and fruits). A recent study found that mutation in *CYP706A3*, a clustered terpene synthase and a cytochrome P450 encoding gene, suppressed sesquiterpene and monoterpene emissions in *Arabidopsis* flowers and changed the floral microbial OTUs in the genus *Pseudomonas* (Boachon et al., 2019a). This study suggested that the *CYP706A3*-generated soluble metabolites played a role in the assembly of specific bacterial taxa colonizing *Arabidopsis* flowers. Furthermore, the floral microbiome may contribute to VOC productions, thereby inducing or reducing the emission of VOCs, and potentially catabolize others. The results suggested that bacteria belonging to the genera *Staphylococcus*, *Bacillus*, and *Sphingomonas* could exploit certain plant VOCs as carbon source, which may reduce their emission rates (Helletsgruber et al., 2017). These bacterial groups contribute to floral scent differences among flowers. One study found that in bee-pollinated *Penstemon digitalis*, the nectar volatile linalool could slow the growth of bacteria across the *P. digitalis* phyllosphere (Burdon et al., 2018). Another recent study showed that β-caryophyllene in tomato leaves can act as a signature VOC, which can lead to the release of a large amount of salicylic acid (SA) from adjacent tomato roots, and contribute to their similar rhizosphere microbial communities (up to 69%) (Kong et al., 2020). Together, these studies show that the plant VOC-microbiome interactions are widespread and are of great ecological interests. A recent review provided an excellent account of the interaction between the phyllosphere or rhizosphere microbiomes and plant VOC emissions (Schenkel et al., 2019).

### Phytohormones Modulate the Plant Microbiome

Phytohormones are an integral part of the plant defense system, commonly known as the plant’s systemic acquired resistance (SAR) and induced systemic resistance (ISR). Phytohormones are a class of small bioactive molecules (Chen X. et al., 2020). In addition to regulating plant physiological and morphological responses, phytohormones also impact the plant microbiome. Phytohormones known to influence plant microbiomes include SA, jasmonic acid (JA), ethylene (ET), abscisic acid (ABA), and strigolactones (SL). Below we briefly review these findings.

The first study on the potential effect of SA on the phyllosphere microbiome examined an SA mutant of *Arabidopsis thaliana* and found limited difference in the phyllosphere microbiome between the SA mutant and the wild-type (Bodenhausen et al., 2014). However, a different study found SA to be capable of modulating the root microbiome of *A. thaliana* (Lebeis et al., 2015). Specifically, plants with altered SA signaling had root microbiomes that differed from each other in their relative abundance of Proteobacteria, Koribacteraceae, Intrasporangiaceae, Kineosporiaceae, Micromonosporaceae, Nocardioidaceae as well as the core microbiome when compared with those of wild-type plants. The study further showed that different bacterial strains responded to SA in different ways, either as a growth signal or as a carbon source, which in turn affected the root microbiome. While the induction of SA-mediated defenses reduced endophytic bacterial community diversity in *Arabidopsis* (Kniskern et al., 2007), certain members of the endophytic microbiome showed evidence of SA-related dependence for successful colonization. One study showed that in wheat, SA caused changes in microbiome through allelopathy (Kong et al., 2018).

Similarly, the effects of JA on plant microbiome are also evident. One study suggested that JA signaling was involved in controlling the density of *Azoarcus* endophyte, thereby shaping the beneficial microbiome in rice roots (Chen X. et al., 2020). The activation of JA-dependent defense mechanisms led to suppression of the SA-mediated defenses against the hemi biotrophic pathogen *P. syringae pv. tomato* (Wasternack and Hause, 2013). However, the addition of external methyl-JA also affected the root microbiome of *A. thaliana* (Carvalhais et al., 2013) and wheat (Liu et al., 2017). Here, JA acted as a SAR inducer in leaves to impact both the phyllosphere and endophytic microbiomes. Apart from JA, derivatives of JA are also capable of influencing the plant root microbiome (Carvalhais et al., 2017; Sasse et al., 2018). Compared with the wild-type, *Arabidopsis* mutants with JA signaling pathway defects showed lower amounts of asparagine, ornithine, and tryptophan, as well as increased abundance of *Streptomyces*, *Bacillus*, *Enterobacteriaceae*, and *Lysinibacillus* taxa, in the root microbiome (Carvalhais et al., 2015). A study in 2007 showed that plants deficient in JA-mediated defenses had greater epiphytic bacterial diversity (Kniskern et al., 2007).

In addition to JA and SA, ethylene (ET), another phytohormone, can also diffuse through air- and water-filled pores in the soil (Broekgaarden et al., 2015). Similar to SA and JA, ET can modulate arbuscular mycorrhizal colonization and root nodulation in legume-rhizobia symbioses (Nascimento et al., 2018). Therefore, like many VOCs, ET has a wide effective range in soil, including mediating long-distance attraction of bacteria to roots. For example, studies on ET mutants showed that mutations in the *ein2* gene altered rhizosphere microbiome (Doornbos et al., 2011). A recent study also suggested that ET production in peanut roots induced by cyanide could alter the microbiome and re-assembled the microbial co-occurrence network of peanuts by increasing the abundance of *Catenulispora* sp., a keystone actinobacterium, in the intercropped peanut rhizosphere (Chen Y. et al., 2020). While the mechanism of how ET works in mediating the plant microbiome is not known, one study suggested that glucosinolate might be involved (Pangesti et al., 2016).

Different phytohormones induce different effects on plant microbiomes. ABA is a common phytohormone and the exogenous application of ABA caused a preferential selection for microbes in the genera *Limnobacter*, *Massilia*, and *Cellvibrio* in a potting soil mixture (Carvalhais et al., 2014). Strigolactones (SL) are commonly exuded from roots under phosphate or nitrogen starvations to attract AM fungi, and their biosynthesis are downregulated after colonization of AM fungi. In contrast, SA, ET, and gibberellins (GA) can all inhibit both AM and root nodule symbiosis (Rodriguez et al., 2019). A recent study found that SL-mediated metabolic pathways are likely involved in the regulation of root microbiome in rice. In SL deficient mutants, there was a higher bacterial richness and a lower fungal diversity than the wild-type plants (Nasir et al., 2019). In addition, certain beneficial bacteria, including those in Nitrosomonadaceae and Rhodanobacter, were significantly decreased in SL mutants compared to the WT (Nasir et al., 2019). Two recent reviews summarized the relevant research progress of ABA and SL as regulators of plant–microbiome interactions (Shtark et al., 2018; Nasir et al., 2020).

Plant microbiomes contain many beneficial and pathogenic microbes. Overtime, plants have evolved mechanisms to recognize these microbes and correspondingly reprogram their defenses to enable or limit the colonization of specific microbes (Zhou et al., 2020). Apart from the pathways specific for phytohormones, the different phytohormone signaling pathways are interconnected at the molecular and phenotypic levels (Rodriguez et al., 2019). Some of the phytohormones act antagonistically with each other, potentially enabling certain microbes to exploit such antagonism to evade host defense and facilitate their own colonization (Jha et al., 2018). During this dynamic interactions, the plant microbiome may also develop resistance to PSMs (Chen Q.-L. et al., 2020). There is an increasing interest in this expanding field of phytohormone–microbiome interactions for both fundamental and applied research.

### Infected Plants Recruit Beneficial Microbes

Interestingly, plants infected by pathogens may change their root exudates which may serve as signals to recruit beneficial root microbes. For example, upon attack by fluorescent pseudomonads (*P. protegens*), *Ordeum vulgar*e L. selectively recruited the Fluorescent pseudomonads carrying antifungal traits to its root microbiome. Such a recruitment leads to a reduced impact by the pathogen on host plants (Dudenhöffer et al., 2016). The same phenomenon was found in citrus root-associated microbiome change upon infection by Huanglongbing (HLB) (Zhang et al., 2017). In *Arabidopsis*, plants challenged by the foliar pathogen *Pseudomonas syringae* pv. *tomato* (*Pst*) exudated lower levels of sugars, alcohols, and short-chain organic acids (SCOAs) and higher levels of amino acids, nucleotides, and long-chain organic acids (LCOAs). These changes lead to the recruitment of beneficial rhizosphere microbes, including a few in Proteobacteria (Yuan et al., 2018). Similarly, the infection of sugar beets by the wilt fungal pathogen *Rhizoctonia solani* caused the enrichment of several endophytic bacteria belonging to *Chitinophaga*, *Flavobacterium*, and *Pseudomonas* species resulting in an activation of their biosynthetic gene clusters to suppress the fungal pathogen (Carrion et al., 2019). These organisms produce antifungal effectors, including cell wall-degrading enzymes, and secondary metabolites such as phenazines, polyketides, and siderophores, that can contribute to their effects on the root mycobiome.

### Secreted Root Mucilage Shapes the Nitrogen-Fixation Microbiome

A study on Mexican maize found that the mucilage associated with the aerial roots of Sierra Mixe maize *Z. mays* ssp. *mexicana* (teosinte) can feed a complex diazotrophic microbiome. The diazotrophic microbiome includes microbes containing active nitrogenase, and the fixed nitrogen can be efficiently transported from the nitrogen-fixing microbes to host plants (Van Deynze et al., 2018). Interestingly, this mechanism allows maize to fix up to 82% of its nitrogen needs from the atmosphere. The maize mucilage was rich in monosaccharides such as arabinose, fucose, galactose, xylose, glucuronic acid, and mannose. Unlike most modern maize varieties, the Sierra Mixe maize variety can develop extensive aerial roots and secrete large amounts of mucilage after rain (Bennett et al., 2020). The monosaccharides in mucilage is not commonly found in plant cell walls and may select for specific mutualistic, nitrogen-fixing bacteria that are uniquely capable of degrading and consuming the mucilage mono- and poly- polysaccharide in exchange for fixing atmospheric nitrogen to benefit the plants (Amicucci et al., 2019). The study paves the way toward developing innovative strategies for biological nitrogen fixation in cereal plants. Indeed, a model for plant-microbe association capable of supporting diazotrophic activity was proposed to support nitrogen fixation in cereal crops (Bennett et al., 2020). On the other hand, mucilage may contain proteins and other metabolites with antimicrobials that function in defense against fungal and bacterial pathogens (Sasse et al., 2018).

## Plant Microbiomes Contribute to the Productions of PSMs

Previous studies have highlighted the capabilities of plant microbiomes to influence important plant traits, such as growth, abiotic stress tolerance, resistance to infectious diseases, and the synthesis of plant growth promoting (PGP) hormones. At present, our understanding of the effects of the microbiome on PSMs, including their mechanism of action remains quite limited. This is different from the large body of literature showing how PSMs can shape the plant–microbiome structure. Improved understanding of PSMs profiles could be achieved by investigating the interaction of the plant (especially medicinal plants and economic crops) with their microbiomes. According to a recent study by Finkel et al. (2020), bacteria in the genus *Variovorax* manipulated *Arabidopsis* root growth and host plant auxin and ethylene levels to influence the development of the *Arabidopsis* root.

### Microbiomes Contribute to Productions of PSMs in Medicinal Plants

For medicinal plants, investigations have shown that the plant microbiomes could influence host plants’ productivity of important medicinal components such as alkaloids, steroids, terpenoids, etc. For example, two recent studies indicated that plant–microbiome interactions could improve biomass production of *Salvia miltiorrhiza* and influence tanshinone production, which is the major class of bioactive medicinal components from this plant (Chen et al., 2018; Huang et al., 2018). In this study, *S. miltiorrhiza* possesses a distinctive seed-associated microbiome, including *Pantoea*, *Pseudomonas*, *Sphingomonas*, and Dothideomycetes. This microbiome contains a gene reservoir related to the synthesis of forterpenoid backbone and other compounds, thus providing additional metabolic capabilities to host plants (Chen et al., 2018). Another study suggested that *Echinacea purpurea* is an excellent model for studying medicinal plant–microbiome interactions (Maggini et al., 2020). The *E. purpurea* microbiome (bacterial strains isolated from stems and leaves) interaction model showed that microbiome can affect the production of VOCs, phenylpropanoid, and alkamides in the plants (Maggini et al., 2017, 2019a,b).

### Microbiome Contributes to Functional PSMs of Economic Crops and Other Plants

Aside from medicinal plants, other economic crops, such as *Cannabis sativa*, are attractive host plants to investigate plant–microbiome interactions. *Cannabis sativa* produces many functional secondary metabolites. Recent research showed that the endophytic bacteria (plant growth-promoting bacteria, PGPR) consortium within *C. sativa* included *Azospirillum brasilense*, *Gluconacetobacter diazotrophicus*, *Burkholderia ambifaria*, and *Herbaspirillum seropedicae.* These endophytic microbes facilitated the growth and development of *Cannabis* and the accumulation of Δ9-tetrahydrocannabinol (THC) and cannabidiol (CBD) (Pagnani et al., 2018; Taghinasab and Jabaji, 2020).

Similarly, inoculation of *Papaver somniferum* L. with a consortium of *Marmoricola* sp. and *Acinetobacter* sp. increased the morphine yield by enhancing the expression of *COR*, a key gene for morphine biosynthesis (Ray T. et al., 2019). In addition, three fungal endophytes (*Fusarium redolens*, *Phialemoniopsis cornearis*, and *Macrophomina pseudophaseolina*) were found to regulate forskolin biosynthesis in *Coleus forskohlii* (Mastan et al., 2019). Plants can also detect certain molecules released by microbiomes through a chemical recognition system, which can subsequently trigger plants to generate signal transduction networks and make corresponding changes in related gene activities, and leading to the accumulation of certain PSMs (Tidke et al., 2019). Importantly, horizontal gene transfer (HGT) in plants-endophytes may also lead to changes in plant secondary metabolic products (Wang et al., 2019). One recent study showed that local colonization of roots by bacteria in the genus *Bacillus* triggered systemic exudation of acylsugars SMs in tomato (Korenblum et al., 2020). Both leaf and root metabolomes and transcriptomes changed due to differences in the root microbiome community structure, with different microbiomes inducing specific changes in tomato root exudation, a process called the systemically induced root exudation of PSMs (SIREM) (Korenblum et al., 2020). However, the underlying molecular mechanisms of functional PSMs synthesis regulated by microbiome- have not been completely elucidated.

### Microbiomes Influence Plant VOCs

Plant microbiomes can participate in and/or influence the production of plant VOCs. For example, the suppression of phyllospheric microbiome in *Sambucus nigra* by antibiotic fumigation also changed the composition and proportion of terpenes in the volatile mix (Peñuelas et al., 2014). This result was confirmed in a later research showing that the application of antibiotics decreased the concentration of acetyl-CoA, citraconic acid, isoleucine, and several other PSMs (such as terpenes and phenols in the epiphytic extracts) in the same plant *S. nigra* (Gargallo-Garriga et al., 2016). Similar observations were made in *Penstemon digitalis* (Burdon et al., 2018), *Arabidopsis thaliana* (Raza et al., 2020), *Brassica rapa* (Helletsgruber et al., 2017), petunia (Boachon et al., 2019b), and *Atractylodes lancea* (Zhou et al., 2018). A recent review provided an excellent summary on the effects of plant microbiomes on plant VOC emissions (Schenkel et al., 2019).

### Are Secondary Metabolites From Plants or Their Microbiomes?

The subsections above discussed how the plant microbiome could contribute to host PSMs. However, it is entirely possible that some of these so-called “PSMs” could be the biosynthetic products of their plant microbiome, including those by their endophytic microbes. Endophytes can produce diverse classes of phytochemicals, including podophyllotoxin, paclitaxel (taxol), deoxypodophyllotoxin, and camptothecin that are also produced by plants (Etalo et al., 2018b; Furtado et al., 2019; Mastan et al., 2019). A previous review discussed endophytic microbiome as potential sources of bioactive compounds (Ray S. et al., 2019). It is necessary to distinguish which of these compounds are produced by host plants and which ones by the plant microbiome. Such knowledge will help with novel developments in the *in situ* analysis of metabolites during the interaction between plants and microbes.

Aside from produce secondary metabolites similar to those produced by plants, endophytes can metabolize secondary compounds produced by host plants. For example, the leaf endophytic mycobiome could metabolize glycosylated flavonoids, the secondary metabolome of *Cephalotaxus harringtonia* (Tian et al., 2014). In another example, deglycosylated flavonoids showed beneficial effects on the hyphal growth of their endophytic fungi. Similarly, the biotransformation of *Huperzine* has also been found in fungal endophytes of *Huperzia serrata* (Zhan et al., 2019). Two recent reviews summarized microbiome-induced metabolic changes in roots and shoots of various crop species (Korenblum and Aharoni, 2019; Ray S. et al., 2019).

### Microbial Secondary Metabolites (MSMs) Influence Plant Traits

While the focus of this review is on how PSMs impact plant microbiomes and how the plant microbiomes can influence the production of PSMs, there have been extensive documentations of how microbial secondary metabolites (MSMs) can impact plant growth and development. Here we describe a few examples. On the one hand, many plant pathogenic microbes can secrete toxins that cause diseases and death to plants. For example, sphinganine-analog mycotoxins including fumonisins and AAL-toxins produced by plant pathogenic fungi in the *Fusarium* genus and in *Alternaria alternata* f. sp. Lycopersici respectively have diverse cytotoxicity and phytotoxicity and are a destructive force to crop production worldwide (Chen J. et al., 2020). On the other hand, there are many examples of bacteria and fungi that produce plant growth – promoting SMs. For example, rhizobacterium *Bacillus tequilensis* SSB07 produces several phytohormones including gibberellins (GA1, GA3, GA5, GA8, GA19, GA24, and GA53), indole-3-acetic acid, and ABA. Application of *B. tequilensis* SSB07 enhanced the growth of Chinese cabbage seedlings and increased the shoot length and biomass, leaf development, and photosynthetic pigment contents of soybeans. For *B. tequilensis* SSB07, its plant growth-enhancing effects were further increased under heat stress, by significantly upregulating the endogenous JA and SA contents in the soybean phyllosphere while down-regulating the production of stress-responsive ABA (Kang et al., 2019).

The positive effects of MSMs on plant growths are shown not only for agricultural crops and vegetables but also for trees. For example, two bacterial strains, *Bacillus* sp. s50 and *Paenibacillus* sp. s37, recently showed significant effects on *Abies nordmanniana*, the most common Christmas tree species in the world. Both bacteria produced high quantities of indole-3-acetic acid, with *Bacillus* sp. s50 increased the seed germination rate and systemic resistance to pathogens while *Paenibacillus* sp. s37 increased plant root growth in both greenhouse and field conditions (Garcia-Lemos et al., 2020). Similar to those found in PGP rhizobacteria, several fungal species such as *Trichoderma virens* are also known to produce indole-3-acetic acid and other auxin-related compounds to positively impact the growth and development of plants, including rice, cotton, and *Arabidopsis* (Contreras-Cornejo et al., 2009).

Aside from phytohormones, the plant microbiomes can also produce abundant VOCs that can impact plant phenotypes (Kai et al., 2009). Many VOCs have been reported from the plant microbiome, including alcohols, aldehydes, ammonia, acids, ketones, esters, and terpenes. These microbial VOCs can influence plant communications, participate in defense against pathogens, and promote plant growth and development (Ortíz-Castro et al., 2009). For example, VOCs emitted by the *Bacillus subtilis* GB03 can trigger changes in major hormonal signaling networks in *A. thaliana* and impact the expressions of over 600 genes related to cell wall modifications, primary and secondary metabolisms, stress responses and auxin homeostasis (Zhang et al., 2007). The VOCs emitted by strain *B. subtilis* GB03 included short-chained alcohols, aldehydes, acids, esters, ketones, hydrocarbons, and sulfur-containing compounds and these VOCs increased photosynthetic efficiency and chlorophyll content in *A. thaliana*. Overall, many microbial VOCs analyzed so far showed evidence of not only impacting plants directly but also indirectly such as by regulating the activities of herbivorous insects and plant parasitic nematodes (Hansen and Moran, 2014; Zhang et al., 2020).

## PSMs–Microbiome Interactions Impact Crop Breeding, Abiotic Stress Response, and Plant Invasion

Plant hosts and their microbiome are highly interlinked and may have co-evolved to function as a meta-organism or holobiont with integrated ecologies. In domesticated crops (predominantly selected for yield traits), microbiomes can also be domesticated, causing different cultivars to be preferentially associated with different microbiomes (Escudero-Martinez and Bulgarelli, 2019). A number of studies suggested that crop microbiomes may have been affected by the domestication process in several crops, including barley (Bulgarelli et al., 2015), rice (Edwards et al., 2018), and the common bean (Perez-Jaramillo et al., 2019). These studies revealed the differences in plant microbiomes between modern cultivars and their wild ancestors in these species. Further studies identified that domestication changed root exudates and several secondary metabolites in modern varieties, likely contributing to the recruitment and maintenance of the plant microbiomes (Iannucci et al., 2017). The findings on PSMs–microbiome interactions have provided valuable insights to guide microbiome-based approaches to improve agricultural productivity. Given the large species diversity and enormous number of potential interactions between PSMs and microbes within individual plants, we are far from understanding the biology of the plant system and its microbiome (or PSMs and microbiome). Identifying specific variations in root exudation among plant species and genotypes could suggest the potential for manipulation of root exudation or PSMs in agricultural cultivars, in order to create specific selective effects on the plant microbiome (Bakker et al., 2012).

Despite many studies on abiotic stress tolerance of crop species, responses of roots to such stresses have so far largely been overlooked. A recent review indicated that plant-associated microbiomes can influence several plant traits including growth and abiotic stress tolerance (de Vries et al., 2020). Similarly, host plants also can adapt to changing environments by adjusting their production of PSMs (Bont et al., 2020). Indeed, interests in PSMs have been significantly enhanced with the knowledge of its importance in enhancing abiotic stress tolerance (de Vries et al., 2020), plant nutrient uptake, and the formation of humus in soil (Sokol et al., 2019). Such studies suggest that PSMs may be central to ecosystem responses to abiotic stresses and that we need an integrated approach to enhance the joint plant-microbiome responses to stresses. On the one hand, PSMs through root exudates can be abiotic stress response mediators. For example, changes in root exudates can help recruit microbiome associations to improve nutrient and water retentions (Huang et al., 2017), and to reduce damaging reactive oxygen species (ROS) by increasing the production of root peroxidases (Naylor and Coleman-Derr, 2017). The exudates of *Quercus ilex* under drought stress contained primarily SMs (71% of total metabolites) (Gargallo-Garriga et al., 2018), brought about mainly by regulating the expression of genes involved in secondary metabolite biosynthesis (Xu et al., 2018; Varoquaux et al., 2019). The altered PSMs further influence the structure of root microbiome, including the recruitment of Actinobacteria, *Streptomyces* or Firmicutes, contributing to the plants’ DefenseBiome and enhancing the plants’ survival under conditions (Bakker P. A. H. M. et al., 2018; Liu and Brettell, 2019; Liu H. et al., 2020; Williams and de Vries, 2020). Together, these studies suggested that root exudates could serve as signals to reshape root microbiome, by acting as chemoattractant or nutrition sources to reconstruct microbiomes to help alleviate abiotic stresses on host plants. At present, the exact chemicals that promote such relationships remain largely unknown. Deciphering this interaction could advance our ability to use microbiome to enhance abiotic stress tolerance in crop plants (Cheng et al., 2019; de Vries et al., 2020).

Invasive plants can change element cycling, soil nutrient pools, and/or soil microbiome that can all potentially accelerate further invasion and prevent re-establishment of native species (Stefanowicz et al., 2017). An example is the invasive plant *Ageratina adenophora* that changed the local soil microbial community and further enhanced *A. adenophora*’s competitive advantage over native plants (Chen L. et al., 2019). The detection and allelopathy of plant neighbors are driven by signal chemicals secreted by roots. There have been many studies on the role of below-ground function of PSMs-microbiome, such as plants releasing SMs (also including VOCs) to communicate with their root microbiome to gain a competitive advantage over other plants (Schandry and Becker, 2020). An example of PSM attracting beneficial microbes was shown in maize with exudate Benzoxazinoids attracting plant-beneficial *Pseudomonas* strains to the maize rhizosphere (Ahmad et al., 2011; Cotton et al., 2019). Another study supported a scenario in which an invasive plant, the Chinese tallow tree (*Triadica sebifera*), enhanced its AMF association and invasion success by changing its root flavonoid metabolism (Pei et al., 2020). Similar findings have been reported from thistle (Verbeek and Kotanen, 2019), *Spartina alterniflora* (Yang et al., 2019), and others (Kamutando et al., 2019; Pei et al., 2020). The recruited microbiome of invasive plants could directly or indirectly interfere their antagonism toward other plants via SMs, enhancing host plant nutrient acquisition (phosphorous and nitrogen) and modulating host root physiology (such as root exudation). Many crop species (including wheat, maize, and rice) are allelopathic, thus, targeted exploitation of allelopathy among plants to reduce weed invasion coupled with a simultaneous reduction of herbicide application provides an attractive option for sustainable agriculture (Schandry and Becker, 2020). For example, investigating model SynComs with various PSMs-microbiome strategies can help derive methods to suppress parasitic weeds in agricultural field. Such methods hold great promise for developing novel integrated crop management strategies (Masteling et al., 2019). However, although some PSMs such as several VOCs are among the biochemically best-characterized allelochemicals, the extent and the molecular mechanisms by which the release of PSMs influencing the root microbiome requires further investigation.

Phosphate is a limiting nutrient in most crop fields. However, the effects of phosphate on microbiome have not been fully described. At present, there are two opposing types of results. In the first, microbes recruited by the PSMs under phosphate limitation provide the plants an advantage in coping with phosphate limitation (Castrillo et al., 2017; Fabiańska et al., 2019; You et al., 2020). On the other hand, the microbes could extract the limited amount of phosphate from the soil and make the phosphate less available to plants (Finkel et al., 2019). Together, these results suggest that different plant-microbiome combinations likely react to phosphate limitations differently. Further research is needed to understand how PSMs might be involved in mediating the plant–microbiome interactions for individual species during phosphate starvation.

## Discussion and Future Prospects

While studies on the human (gut) microbiome have stolen most of the limelight, botanists have also been making progress toward elucidating the composition and function of plant microbiomes and PSMs over the last few years. In spite of a large number of contributions on plant microbiome, a thorough comprehension on plant microbiome structure, dynamics, and function associated with PSMs still remains largely unexplored. One potential area of research involves understanding the factors influencing plant microbiome assembly, and the signaling crosstalk in plant–microbiome interactions (Sasse et al., 2018). On the one hand, systematic research on the associated microbiomes in model plants, such as *Arabidopsis*, rice and maize, could help clarify the roles microbes may play in modulating the biosynthetic pathways of PSMs. Approaches such as SynCom may provide both functional and mechanistic insights into how plants regulate their microbiomes, and on how the microbiomes influence PSMs. Secondly, the single-cell genomics and specialized plant metabolome analytical tools are opening new possibilities for a diversity of potential research topics. Thirdly, spatial patterns of PSMs–microbiome interactions are largely unexplored. To improve the root exudate analysis, several modern technologies such as microfluidics and bacterial biosensors that respond to selected PSMs have been introduced (Massalha et al., 2017). And last but not the least, the underpinning genetic controls on PSMs and how they are affected by changing microbiomes and environmental conditions require greater focus.

### Methods for Detecting and Tracking Plant Secondary Metabolites

The focus of this review is on the interaction between PSMs–microbiome. Thus, it’s important to discuss methods for plant metabolome analysis. Metabolome analyses include data acquisition and processing. Data processing includes data normalization, peak alignment, and data scaling (Duan and Qi, 2015; Tahir et al., 2019). Several excellent software and websites are currently available for the processing of NMR and MS data. These include MZmine 2, XCMS, Open MS, Decon2LS, and MS-DIAL, all of which have been extensively used for diverse sets of metabolomics data. Misra (2020) provided a recent review that summarized over 95 metabolomics tools, software, and database.

In addition, the fine-scale dynamics between the PSMs-microbiome are of special importance to improve our understanding on plant–microbe interactions. Indeed, there is a growing interest in tracing and narrowing PSMs analyses down to single-cell level, which will be helpful to observe in-situ metabolism and trace metabolites in plant–microbiome interaction. Sensitive detection technologies and innovative cell-sampling techniques are needed to profile and trace metabolites in single cells. However, the field is still in its infancy for plant research. As PSMs are accumulated due to the activities of both host plant and its microbiome, strategies that allow metabolite traceability should be used to elucidate the origin of metabolites and to interpret their actions. The studies of the intestinal microbiome from humans and animals provide good references from which to design similar studies on plant–microbiome interactions (Koh et al., 2018; Duncan et al., 2019; Nemet et al., 2020).

One approach called Exometabolomics could provide novel insights into root microbiome. This approach investigates the root-derived compounds as carbon sources consumed by individual microbial strains and identifies substrate preferences of individual microbes from a mixture of exuded metabolites (Jacoby et al., 2018). Unfortunately, tracking the dynamics of root–microbiome interactions at high spatial resolution is still time consuming and requires significant expertise. Other methods include a microfluidics-based approach termed tracking root interactions system (TRIS) (Massalha et al., 2017) and a root-microbe interaction chip (RMI-Chip) (Noirot-Gros et al., 2020). These biosensors (Pini et al., 2017) or microfluidics (Millet et al., 2019) based methods enable direct imaging of root–microbiome interactions in real time, and provide spatiotemporal non-destructive analysis of samples *in situ* (Lenzewski et al., 2018). On the other hand, methods for whole-sample metabolic profiling of non-sterile rhizosphere soil have also been explored (Petriacq et al., 2017). These novel approaches thus allow researchers to investigate microbiome substrate preference for a number of metabolites at the same time, mimicking the real PSMs–microbiome interactions.

### Bioinformatics of Plant Metabolome–Microbiome Interactions

Aside from the development in hardware for data acquisition, software that integrates information from both the metabolome and the microbiome is also needed. For example, statistical methods for metabolome-microbiome data integration have been developed to identify the potential molecular markers driving their interactions (Lamichhane et al., 2018). Unfortunately, though improvements have been made, complete annotation of plant metabolomes is not yet possible (Lucaciu et al., 2019). Furthermore, there is a lack of in-depth understanding on how to integrate multi-omics data such as those from the proteome and transcriptome. The development of more reliable bioinformatics tools such as neural networks method is also urgent.

Despite these potential problems, recent studies suggested that untargeted metabolomics datasets showed a closer correlation with the microbiome data than those of targeted approaches, especially when they are compared with specific microbial metabolites (Melnik et al., 2017; Lamichhane et al., 2018). At present, several metabolite analysis methods are available and their use depends mainly on study objectives. These metabolic profiling methods include nontargeted metabolomics, widely targeted metabolites, metabolite target analysis (targeted), and metabolite fingerprinting (Tahir et al., 2019). A new integrated method named ESI-triple quadrupole-linear ion trap (Q TRAP)-MS (Luo et al., 2016) or ESI-QqTOF-MS (Chen et al., 2013) for large-scale detection, identification, and quantification of common metabolites has also been developed (Duncan et al., 2019; Kozuka et al., 2020). However, there is no specialized database for plant microbiome–metabolome information that is linked to environmental conditions (Lucaciu et al., 2019). Priority needs to be given to the development of such databases for functional interpretation of the increasingly common large-scale multi-omics plant microbiome data.

### Confirming Causal Relationship Between PSMs–Microbiome Interactions Using Synthetic Communities

Both the plant microbiome and PSMs play important roles in plant health, impacting agriculture and food security (Haney et al., 2015). Though progresses have been made in our understanding of their interactions, many questions remain. For example, which special microbiome was attracted by PSMs and how to maintain the activities and abundance of PSMs? How do PSMs discriminate beneficial microbiome from pathogenic ones? Future research efforts should be devoted to understanding the modes with which microbiome affects PSMs in various plant tissues, evaluating the direction and magnitude of changes in microbiome as mediated by PSMs. Similarly, understanding how changes in PSMs are affected by the plant microbiomes is also important. A promising approach to understand reciprocal effects of plants and their microbiota is through SynCom, using sequenced and cultivated bacteria to provide simple and reproducible systems to study PSMs–microbiome interactions (Durán et al., 2018; Liu et al., 2019). Such a system allows precise variations in stress levels, exposure to infectious agents, phytohormone concentrations and metabolism, nutrition supply, etc. (Koprivova et al., 2019; Liu H. et al., 2020). Another study developed a machine learning computational approach to design SynCom, making it possible to infer causal relationships between microbiome membership and host plant phenotypes, potentially allowing the design of novel communities (Herrera Paredes et al., 2018). In addition, SynComs can be further combined with PSMs detection technologies. In summary, SynCom systems can provide crucial insights into the two-way interactions between PSMs and plant microbiome.

### Connecting PSMs–Microbiome Relationships With Plant Breeding

Plant scientists are beginning to consider the plant microbiome as plants’ “secondary genome” that can provide host plants with microbe-derived metabolites and traits. During traditional crop breeding, breeders select traits for yield or nutrition but not for PSMs-microbiome relationships. However, as shown above, microbiomes can respond rapidly to changes in PSMs such as those in root exudates and in the phyllosphere. Consequently, the plant microbiome could be treated as a selectable trait during breeding that could be manipulated chemically through PSMs. A pre-requisite for success in such a breeding program is a broad understanding of the relationships and changes of PSMs and microbiome during crop domestication. As described above, domestication can modify PSMs – microbiome relationship. Furthermore, previous studies have shown that different corps attract different microbiomes and that the microbiomes can have different effects on different host plants. A recent review proposed using desirable microbiomes as selective markers to identify potential beneficial microbiome for specific crop varieties (Pascale et al., 2020). In this proposal, PSMs play a key role in the selection process, by attracting various beneficial microbes and/or repelling detrimental one.

At present, in-depth and systematic investigations on the effects of the PSMs and functional microbiome on economic crops are relatively limited. Indeed, the function and dynamics of PSMs-functional microbiome interactions remain unexplored in most economic crops. Some economic crops, such as garlic and ginger, can be widely used as models to analyze such interactions and to enhance the production of the desirable metabolites such as allicin and curcumin for commercial purposes. Indeed, understanding the relationship between economic crop PSMs and functional microbiome can lead to improved agricultural practices that enhance plant fitness and increase the yield of functional secondary metabolites.

## Author Contributions

YC and JX conceived, structured, and finalized the manuscript. ZP coordinated the literature research and drafted the initial version of the manuscript. All authors contributed to the literature search and reviewing and finalizing the manuscript.

## Conflict of Interest

The authors declare that the research was conducted in the absence of any commercial or financial relationships that could be construed as a potential conflict of interest.

## References

[B1] Ahmad S. Veyrat N. Gordon-Weeks R. Zhang Y. Martin J. Smart L. (2011). Benzoxazinoid metabolites regulate innate immunity against aphids and fungi in maize. *Plant Physiol.* 157 317–327. 10.1104/pp.111.180224 21730199 PMC3165881

[B2] Amicucci M. J. Galermo A. G. Guerrero A. Treves G. Nandita E. Kailemia M. J. (2019). Strategy for structural elucidation of polysaccharides: elucidation of a maize mucilage that harbors diazotrophic bacteria. *Anal. Chem.* 91 7254–7265. 10.1021/acs.analchem.9b00789 30983332

[B3] Bakker M. G. Manter D. K. Sheflin A. M. Weir T. L. Vivanco J. M. (2012). Harnessing the rhizosphere microbiome through plant breeding and agricultural management. *Plant Soil.* 360 1–13. 10.1007/s11104-012-1361-x

[B4] Bakker O. B. Aguirre-Gamboa R. Sanna S. Oosting M. Smeekens S. P. Jaeger M. (2018). Integration of multi-omics data and deep phenotyping enables prediction of cytokine responses. *Nat. Immunol.* 19 776–786. 10.1038/s41590-018-0121-3 29784908 PMC6022810

[B5] Bakker P. A. H. M. Pieterse C. M. J. de Jonge R. Berendsen R. L. (2018). The soil-borne legacy. *Cell* 172 1178–1180. 10.1016/j.cell.2018.02.024 29522740

[B6] Battin T. J. Besemer K. Bengtsson M. M. Romani A. M. Packmann A. I. (2016). The ecology and biogeochemistry of stream biofilms. *Nat. Rev. Microbiol.* 14 251–263. 10.1038/nrmicro.2016.15 26972916

[B7] Bennett A. B. Pankievicz V. C. S. Ane J. M. (2020). A model for nitrogen fixation in cereal crops. *Trends Plant Sci.* 25 226–235. 10.1016/j.tplants.2019.12.004 31954615

[B8] Bhattacharjee A. Velickovic D. Wietsma T. W. Bell S. L. Jansson J. K. Hofmockel K. S. (2020). Visualizing microbial community dynamics via a controllable soil environment. *Msystems* 5:e645-19. 10.1128/mSystems.00645-19 PMC7018529 32047062

[B9] Boachon B. Burdloff Y. Ruan J.-X. Rojo R. Junker R. R. Vincent B. (2019a). A promiscuous cyp706a3 reduces terpene volatile emission from *Arabidopsis* flowers, affecting florivores and the floral microbiome. *Plant Cell* 31 2947–2972. 10.1105/tpc.19.00320 31628167 PMC6925022

[B10] Boachon B. Lynch J. H. Ray S. Yuan J. Caldo K. M. P. Junker R. R. (2019b). Natural fumigation as a mechanism for volatile transport between flower organs. *Nat. Chem. Biol.* 15 583–588. 10.1038/s41589-019-0287-5 31101916

[B11] Bodenhausen N. Bortfeld-Miller M. Ackermann M. Vorholt J. A. (2014). A synthetic community approach reveals plant genotypes affecting the phyllosphere microbiota. *PLoS Genet.* 10:e1004283. 10.1371/journal.pgen.1004283 PMC3990490 24743269

[B12] Bont Z. Züst T. Arce C. C. M. Huber M. Erb M. (2020). Heritable variation in root secondary metabolites is associated with recent climate. *J. Ecol.* 108 2611–2624. 10.1111/1365-2745.13441

[B13] Boughton B. A. Thinagaran D. (2018). “Mass spectrometry imaging (msi) for plant metabolomics,” in *Plant Metabolomics: Methods and Protocols*, ed. António C. (New York, NY: Springer). 10.1007/978-1-4939-7819-9_17 29761443

[B14] Brader G. Compant S. Mitter B. Trognitz F. Sessitsch A. (2014). Metabolic potential of endophytic bacteria. *Curr. Opin. Biotechnol.* 27 30–37. 10.1016/j.copbio.2013.09.012 24863894 PMC4045207

[B15] Broekgaarden C. Caarls L. Vos I. A. Pieterse C. M. Van Wees S. C. (2015). Ethylene: traffic controller on hormonal crossroads to defense. *Plant Physiol.* 169 2371–2379. 10.1104/pp.15.01020 26482888 PMC4677896

[B16] Bulgarelli D. Garrido-Oter R. Münch, Philipp C. Weiman A. Dröge J. (2015). Structure and function of the bacterial root microbiota in wild and domesticated barley. *Cell Host Microbe.* 17 392–403. 10.1016/j.chom.2015.01.011 25732064 PMC4362959

[B17] Burdon R. C. F. Junker R. R. Scofield D. G. Parachnowitsch A. L. (2018). Bacteria colonising penstemon digitalis show volatile and tissue-specific responses to a natural concentration range of the floral volatile linalool. *Chemoecology* 28 11–19. 10.1007/s00049-018-0252-x 29540962 PMC5840241

[B18] Buza T. M. Tonui T. Stomeo F. Tiambo C. Katani R. Schilling M. (2019). Imap: an integrated bioinformatics and visualization pipeline for microbiome data analysis. *BMC Bioinformatics* 20:374. 10.1186/s12859-019-2965-4 PMC6610863 31269897

[B19] Caporaso J. G. Kuczynski J. Stombaugh J. Bittinger K. Bushman F. D. Costello E. K. (2010). Qiime allows analysis of high-throughput community sequencing data. *Nat. Methods* 7 335–336. 10.1038/nmeth.f.303 20383131 PMC3156573

[B20] Carrion V. J. Perez-Jaramillo J. Cordovez V. Tracanna V. de Hollander M. Ruiz-Buck D. (2019). Pathogen-induced activation of disease-suppressive functions in the endophytic root microbiome. *Science* 366 606–612. 10.1126/science.aaw9285 31672892

[B21] Carvalhais L. C. Dennis P. G. Badri D. V. Kidd B. N. Vivanco J. M. Schenk P. M. (2015). Linking jasmonic acid signaling, root exudates, and rhizosphere microbiomes. *Mol. Plant Microbe Interact.* 28 1049–1058. 10.1094/MPMI-01-15-0016-R 26035128

[B22] Carvalhais L. C. Dennis P. G. Badri D. V. Tyson G. W. Vivanco J. M. Schenk P. M. (2013). Activation of the jasmonic acid plant defence pathway alters the composition of rhizosphere bacterial communities. *PLoS One* 8:e56457. 10.1371/journal.pone.0056457 PMC3570460 23424661

[B23] Carvalhais L. C. Dennis P. G. Schenk P. M. (2014). Plant defence inducers rapidly influence the diversity of bacterial communities in a potting mix. *Appl. Soil Ecol.* 84 1–5. 10.1016/j.apsoil.2014.06.011

[B24] Carvalhais L. C. Schenk P. M. Dennis P. G. (2017). Jasmonic acid signalling and the plant holobiont. *Curr. Opin. Microbiol.* 37 42–47. 10.1016/j.mib.2017.03.009 28437665

[B25] Castrillo G. Teixeira P. J. Paredes S. H. Law T. F. de Lorenzo L. Feltcher M. E. (2017). Root microbiota drive direct integration of phosphate stress and immunity. *Nature* 543 513–518. 10.1038/nature21417 28297714 PMC5364063

[B26] Chen H. Wu H. Yan B. Zhao H. Liu F. Zhang H. (2018). Core microbiome of medicinal plant salvia miltiorrhiza seed: a rich reservoir of beneficial microbes for secondary metabolism? *Int. J. Mol. Sci.* 19:672. 10.3390/ijms19030672 PMC5877533 29495531

[B27] Chen J. Li Z. M. Cheng Y. Gao C. S. Guo L. T. Wang T. H. (2020). Sphinganine-analog mycotoxins (SAMs): chemical structures, bioactivities, and genetic controls. *J. Fungi.* 6:312. 10.3390/jof6040312 PMC7711896 33255427

[B28] Chen L. Fang K. Zhou J. Yang Z. P. Dong X. F. Dai G. H. (2019). Enrichment of soil rare bacteria in root by an invasive plant ageratina adenophora. *Sci. Total Environ.* 683 202–209. 10.1016/j.scitotenv.2019.05.220 31132698

[B29] Chen M. X. Wang S. Y. Kuo C. H. Tsai I. L. (2019). Metabolome analysis for investigating host-gut microbiota interactions. *J. Formos. Med. Assoc.* 118 (Suppl. 1), S10–S22. 10.1016/j.jfma.2018.09.007 30269936

[B30] Chen Q. Jiang T. Liu Y. X. Liu H. Zhao T. Liu Z. (2019). Recently duplicated sesterterpene (c25) gene clusters in *Arabidopsis thaliana* modulate root microbiota. *Sci. China Life Sci.* 62 947–958. 10.1007/s11427-019-9521-2 31079337

[B31] Chen Q.-L. Hu H.-W. Zhu D. Ding J. Yan Z.-Z. He J.-Z. (2020). Host identity determines plant associated resistomes. *Environ. Pollut.* 258:113709. 10.1016/j.envpol.2019.113709 31838394

[B32] Chen W. Gong L. Guo Z. Wang W. Zhang H. Liu X. (2013). A novel integrated method for large-scale detection, identification, and quantification of widely targeted metabolites: application in the study of rice metabolomics. *Mol. Plant.* 6 1769–1780. 10.1093/mp/sst080 23702596

[B33] Chen X. Marszałkowska M. Reinhold-Hurek B. (2020). Jasmonic acid, not salicyclic acid restricts endophytic root colonization of rice. *Front. Plant Sci.* 10:1758. 10.3389/fpls.2019.01758 PMC7000620 32063914

[B34] Chen Y. Bonkowski M. Shen Y. Griffiths B. S. Jiang Y. Wang X. (2020). Root ethylene mediates rhizosphere microbial community reconstruction when chemically detecting cyanide produced by neighbouring plants. *Microbiome* 8:4. 10.1186/s40168-019-0775-6 PMC6969408 31954405

[B35] Cheng Y. T. Zhang L. He S. Y. (2019). Plant-microbe interactions facing environmental challenge. *Cell Host Microbe.* 26 183–192. 10.1016/j.chom.2019.07.009 31415751 PMC6697056

[B36] Comeau A. M. Douglas G. M. Langille M. G. I. (2017). Microbiome helper: a custom and streamlined workflow for microbiome research. *mSystems* 2:e127-16. 10.1128/mSystems.00127-16 PMC5209531 28066818

[B37] Compant S. Samad A. Faist H. Sessitsch A. (2019). A review on the plant microbiome: ecology, functions, and emerging trends in microbial application. *J. Adv. Res.* 19 29–37. 10.1016/j.jare.2019.03.004 31341667 PMC6630030

[B38] Contreras-Cornejo H. A. Maciìas-Rodrìguez L. I. Cortés-Penagos C. Loìpez-Bucio J. (2009). Trichoderma virens, a plant beneficial fungus, enhances biomass production and promotes lateral root growth through an auxin-dependent mechanism in *Arabidopsis*. *Plant Physiol.* 149 1579–1592. 10.1104/pp.108.130369 19176721 PMC2649400

[B39] Cotton T. E. A. Petriacq P. Cameron D. D. Meselmani M. A. Schwarzenbacher R. Rolfe S. A. (2019). Metabolic regulation of the maize rhizobiome by benzoxazinoids. *ISME J.* 13 1647–1658. 10.1038/s41396-019-0375-2 30796337 PMC6592824

[B40] Davis N. M. Proctor D. M. Holmes S. P. Relman D. A. Callahan B. J. (2018). Simple statistical identification and removal of contaminant sequences in marker-gene and metagenomics data. *Microbiome* 6:226. 10.1186/s40168-018-0605-2 PMC6298009 30558668

[B41] de Vries F. T. Griffiths R. I. Knight C. G. Nicolitch O. Williams A. (2020). Harnessing rhizosphere microbiomes for drought-resilient crop production. *Science* 368:270. 10.1126/science.aaz5192 32299947

[B42] Dhariwal A. Chong J. Habib S. King I. L. Agellon L. B. Xia J. (2017). Microbiomeanalyst: a web-based tool for comprehensive statistical, visual and meta-analysis of microbiome data. *Nucleic Acids Res.* 45 W180–W188. 10.1093/nar/gkx295 28449106 PMC5570177

[B43] Doornbos R. F. Geraats B. P. Kuramae E. E. Van Loon L. C. Bakker P. A. (2011). Effects of jasmonic acid, ethylene, and salicylic acid signaling on the rhizosphere bacterial community of *Arabidopsis thaliana*. *Mol. Plant Microbe Interact.* 24 395–407. 10.1094/mpmi-05-10-0115 21171889

[B44] Duan L.-X. Qi X. (2015). “Metabolite qualitative methods and the introduction of metabolomics database,” in *Plant metabolomics: Methods and applications*, eds Qi X. Chen X. Wang Y. (Dordrecht: Springer).

[B45] Dubey O. Dubey S. Schnee S. Glauser G. Nawrath C. Gindro K. (2020). Plant surface metabolites as potent antifungal agents. *Plant Physiol. Biochem.* 150 39–48. 10.1016/j.plaphy.2020.02.026 32112998

[B46] Dudenhöffer J.-H. Scheu S. Jousset A. (2016). Systemic enrichment of antifungal traits in the rhizosphere microbiome after pathogen attack. *J. Ecol.* 104 1566–1575. 10.1111/1365-2745.12626

[B47] Duncan K. D. Fyrestam J. Lanekoff I. (2019). Advances in mass spectrometry based single-cell metabolomics. *Analyst* 144 782–793. 10.1039/c8an01581c 30426983

[B48] Durán P. Thiergart T. Garrido-Oter R. Agler M. Kemen E. Schulze-Lefert P. (2018). Microbial interkingdom interactions in roots promote *Arabidopsis* survival. *Cell* 175 973.e14–983.e14. 10.1016/j.cell.2018.10.020 30388454 PMC6218654

[B49] Edgar R. C. (2013). Uparse: highly accurate otu sequences from microbial amplicon reads. *Nat. Methods* 10 996–998. 10.1038/nmeth.2604 23955772

[B50] Edgar R. C. Flyvbjerg H. (2015). Error filtering, pair assembly and error correction for next-generation sequencing reads. *Bioinformatics* 31 3476–3482. 10.1093/bioinformatics/btv401 26139637

[B51] Edwards J. A. Santos-Medellin C. M. Liechty Z. S. Nguyen B. Lurie E. Eason S. (2018). Compositional shifts in root-associated bacterial and archaeal microbiota track the plant life cycle in field-grown rice. *PLoS Biol.* 16:e2003862. 10.1371/journal.pbio.2003862 PMC5841827 29474469

[B52] Escudero-Martinez C. Bulgarelli D. (2019). Tracing the evolutionary routes of plant–microbiota interactions. *Curr. Opin. Microbiol.* 49 34–40. 10.1016/j.mib.2019.09.013 31698159

[B53] Etalo D. W. Díez-Simón C. de Vos R. C. H. Hall R. D. (2018a). “Laser ablation electrospray ionization-mass spectrometry imaging (laesi-ms) for spatially resolved plant metabolomics,” in *Plant Metabolomics: Methods and Protocols*, ed. António C. (New York, NY: Springer). 10.1007/978-1-4939-7819-9_18 29761444

[B54] Etalo D. W. Jeon J. S. Raaijmakers J. M. (2018b). Modulation of plant chemistry by beneficial root microbiota. *Nat. Prod. Rep.* 35 398–409. 10.1039/c7np00057j 29722774

[B55] Fabiańska I. Gerlach N. Almario J. Bucher M. (2019). Plant-mediated effects of soil phosphorus on the root-associated fungal microbiota in *Arabidopsis thaliana*. *New Phytol.* 221 2123–2137. 10.1111/nph.15538 30317641 PMC6519159

[B56] Fakhri S. Moradi S. Z. Farzaei M. H. Bishayee A. (2020). Modulation of dysregulated cancer metabolism by plant secondary metabolites: a mechanistic review. *Semin. Cancer Biol.* 10.1016/j.semcancer.2020.02.007 Online ahead of print 32081639

[B57] Fang C. Fernie A. R. Luo J. (2019). Exploring the diversity of plant metabolism. *Trends Plant Sci.* 24 83–98. 10.1016/j.tplants.2018.09.006 30297176

[B58] Fang H. Huang C. Zhao H. Deng M. (2015). Cclasso: correlation inference for compositional data through lasso. *Bioinformatics* 31 3172–3180. 10.1093/bioinformatics/btv349 26048598 PMC4693003

[B59] Farré-Armengol G. Filella I. Llusia J. Peñuelas J. (2016). Bidirectional interaction between phyllospheric microbiotas and plant volatile emissions. *Trends Plant Sci.* 21 854–860. 10.1016/j.tplants.2016.06.005 27401253

[B60] Faust K. Sathirapongsasuti J. F. Izard J. Segata N. Gevers D. Raes J. (2012). Microbial co-occurrence relationships in the human microbiome. *PLoS Comput. Biol.* 8:e1002606. 10.1371/journal.pcbi.1002606 PMC3395616 22807668

[B61] Finkel O. M. Salas-González I. Castrillo G. Conway J. M. Law T. F. Teixeira P. J. P. L. (2020). A single bacterial genus maintains root growth in a complex microbiome. *Nature* 587 103–108. 10.1038/s41586-020-2778-7 32999461 PMC10329457

[B62] Finkel O. M. Salas-González I. Castrillo G. Spaepen S. Law T. F. Teixeira P. J. P. L. (2019). The effects of soil phosphorus content on plant microbiota are driven by the plant phosphate starvation response. *PLoS Biol.* 17:e3000534. 10.1371/journal.pbio.3000534 PMC6876890 31721759

[B63] Friedman J. Alm E. J. (2012). Inferring correlation networks from genomic survey data. *PLoS Comput. Biol.* 8:e1002687. 10.1371/journal.pcbi.1002687 PMC3447976 23028285

[B64] Furtado B. U. Golebiewski M. Skorupa M. Hulisz P. Hrynkiewicz K. (2019). Bacterial and fungal endophytic microbiomes of salicornia europaea. *Appl. Environ. Microbiol.* 85:e305-19. 10.1128/AEM.00305-19 PMC6581177 31003988

[B65] Garcia K. Delaux P. M. Cope K. R. Ane J. M. (2015). Molecular signals required for the establishment and maintenance of ectomycorrhizal symbioses. *New Phytol.* 208 79–87. 10.1111/nph.13423 25982949

[B66] Garcia-Lemos A. M. Großkinsky D. K. Saleem A. S. Nicolaisen M. H. Roitsch T. Nybroe O. (2020). Identification of root-associated bacteria that influence plant physiology, increase seed germination, or promote growth of the christmas tree species *Abies nordmanniana*. *Front. Microbiol.* 11:566613. 10.3389/fmicb.2020.566613) PMC7705201 33281762

[B67] Gargallo-Garriga A. Preece C. Sardans J. Oravec M. Urban O. Peñuelas J. (2018). Root exudate metabolomes change under drought and show limited capacity for recovery. *Sci. Rep.* 8:12696. 10.1038/s41598-018-30150-0 PMC6107494 30140025

[B68] Gargallo-Garriga A. Sardans J. Pérez-Trujillo M. Guenther A. Llusià J. Rico L. (2016). Shifts in plant foliar and floral metabolomes in response to the suppression of the associated microbiota. *BMC Plant Biol.* 16:78. 10.1186/s12870-016-0767-7 PMC4822282 27048394

[B69] Geier B. Sogin E. M. Michellod D. Janda M. Kompauer M. Spengler B. (2020). Spatial metabolomics of in situ host–microbe interactions at the micrometre scale. *Nat. Microbiol.* 5 498–510. 10.1038/s41564-019-0664-6 32015496

[B70] Gevers D. Kugathasan S. Denson L. A. Vazquez-Baeza Y. Van Treuren W. Ren B. (2014). The treatment-naive microbiome in new-onset crohn’s disease. *Cell Host Microbe.* 15 382–392. 10.1016/j.chom.2014.02.005 24629344 PMC4059512

[B71] Górniak I. Bartoszewski R. Króliczewski J. (2019). Comprehensive review of antimicrobial activities of plant flavonoids. *Phytochem. Rev.* 18 241–272. 10.1007/s11101-018-9591-z

[B72] Greenblum S. Turnbaugh P. J. Borenstein E. (2012). Metagenomic systems biology of the human gut microbiome reveals topological shifts associated with obesity and inflammatory bowel disease. *Proc. Natl. Acad. Sci. U.S.A.* 109 594–599. 10.1073/pnas.1116053109 22184244 PMC3258644

[B73] Guerrieri A. Dong L. Bouwmeester H. J. (2019). Role and exploitation of underground chemical signaling in plants. *Pest Manag. Sci.* 75 2455–2463. 10.1002/ps.5507 31166074 PMC6771575

[B74] Gweon H. S. Oliver A. Taylor J. Booth T. Gibbs M. Read D. S. (2015). Pipits: an automated pipeline for analyses of fungal internal transcribed spacer sequences from the illumina sequencing platform. *Methods Ecol. Evol.* 6 973–980. 10.1111/2041-210x.12399 27570615 PMC4981123

[B75] Haney C. H. Samuel B. S. Bush J. Ausubel F. M. (2015). Associations with rhizosphere bacteria can confer an adaptive advantage to plants. *Nat. Plants* 1:15051. 10.1038/nplants.2015.51 PMC4806546 27019743

[B76] Hansen A. K. Moran N. A. (2014). The impact of microbial symbionts on host plant utilization by herbivorous insects. *Mol. Ecol.* 23 1473–1496. 10.1111/mec.12421 23952067

[B77] Hartman K. van der Heijden M. G. Roussely-Provent V. Walser J. C. Schlaeppi K. (2017). Deciphering composition and function of the root microbiome of a legume plant. *Microbiome* 5:2. 10.1186/s40168-016-0220-z PMC5240445 28095877

[B78] Helletsgruber C. Dötterl S. Ruprecht U. Junker R. R. (2017). Epiphytic bacteria alter floral scent emissions. *J. Chem. Ecol.* 43 1073–1077. 10.1007/s10886-017-0898-9 29134407 PMC5735204

[B79] Herrera Paredes S. Gao T. Law T. F. Finkel O. M. Mucyn T. Teixeira P. (2018). Design of synthetic bacterial communities for predictable plant phenotypes. *PLoS Biol.* 16:e2003962. 10.1371/journal.pbio.2003962 PMC5819758 29462153

[B80] Hill C. J. Lynch D. B. Murphy K. Ulaszewska M. Jeffery I. B. O’Shea C. A. (2017). Evolution of gut microbiota composition from birth to 24 weeks in the infantmet cohort. *Microbiome* 5:4. 10.1186/s40168-016-0213-y PMC5240274 28095889

[B81] Holmer R. Rutten L. Kohlen W. van Velzen R. Geurts R. (2017). “Commonalities in symbiotic plant-microbe signalling,” in *Advances in Botanical Research*, ed. Becard G. (Cambridge, MA: Academic Press).

[B82] Hu L. Robert C. A. M. Cadot S. Zhang X. Ye M. Li B. (2018). Root exudate metabolites drive plant-soil feedbacks on growth and defense by shaping the rhizosphere microbiota. *Nat. Commun.* 9:2738. 10.1038/s41467-018-05122-7 PMC6048113 30013066

[B83] Huang A. C. Jiang T. Liu Y. X. Bai Y. C. Reed J. Qu B. (2019). A specialized metabolic network selectively modulates *Arabidopsis* root microbiota. *Science* 364:eaau6389. 10.1126/science.aau6389 31073042

[B84] Huang W. Long C. Lam E. (2018). Roles of plant-associated microbiota in traditional herbal medicine. *Trends Plant Sci.* 23 559–562. 10.1016/j.tplants.2018.05.003 29802067

[B85] Huang Y. M. Zou Y. N. Wu Q. S. (2017). Alleviation of drought stress by mycorrhizas is related to increased root h2o2 efflux in trifoliate orange. *Sci Rep.* 7:42335. 10.1038/srep42335 PMC5296721 28176859

[B86] Iannucci A. Fragasso M. Beleggia R. Nigro F. Papa R. (2017). Evolution of the crop rhizosphere: impact of domestication on root exudates in tetraploid wheat (*Triticum* turgidum l.). *Front. Plant Sci.* 8:2124. 10.3389/fpls.2017.02124 PMC5733359 29326736

[B87] Ilhan Z. E. DiBaise J. K. Dautel S. E. Isern N. G. Kim Y.-M. Hoyt D. W. (2020). Temporospatial shifts in the human gut microbiome and metabolome after gastric bypass surgery. *NPJ Biofilms Microbiomes.* 6:12. 10.1038/s41522-020-0122-5 PMC7070067 32170068

[B88] Isah T. (2019). Stress and defense responses in plant secondary metabolites production. *Biol. Res.* 52:39. 10.1186/s40659-019-0246-3 PMC6661828 31358053

[B89] Jacoby R. Chen L. Schwier M. Koprivova A. Kopriva S. (2020). Recent advances in the role of plant metabolites in shaping the root microbiome [version 1; peer review: 3 approved]. *F1000Res* 9:F1000FacultyRev-151. 10.12688/f1000research.21796.1 PMC7047909 32148778

[B90] Jacoby R. P. Martyn A. Kopriva S. (2018). Exometabolomic profiling of bacterial strains as cultivated using *Arabidopsis* root extract as the sole carbon source. *Mol. Plant Microbe Interact.* 31 803–813. 10.1094/mpmi-10-17-0253-r 29457542

[B91] Jha P. Panwar J. Jha P. N. (2018). Mechanistic insights on plant root colonization by bacterial endophytes: a symbiotic relationship for sustainable agriculture. *Environ. Sustain.* 1 25–38. 10.1007/s42398-018-0011-5

[B92] Kai M. Haustein M. Molina F. Petri A. Scholz B. Piechulla B. (2009). Bacterial volatiles and their action potential. *Appl. Microbiol. Biotechnol.* 81 1001–1012. 10.1007/s00253-008-1760-3 19020812

[B93] Kamutando C. N. Vikram S. Kamgan-Nkuekam G. Makhalanyane T. P. Greve M. Le Roux J. J. (2019). The functional potential of the rhizospheric microbiome of an invasive tree species, acacia dealbata. *Microb. Ecol.* 77 191–200. 10.1007/s00248-018-1214-0 29948018

[B94] Kang S. M. Khan A. L. Waqas M. Asaf S. Lee K. E. Park Y. G. (2019). Integrated phytohormone production by the plant growth-promoting rhizobacterium *Bacillus* tequilensis SSB07 induced thermotolerance in soybean. *J. Plant Interact.* 14 416–423. 10.1080/17429145.2019.1640294

[B95] Kessler A. Kalske A. (2018). Plant secondary metabolite diversity and species interactions. *Annu. Rev. Ecol. Evol. Syst.* 49 115–138. 10.1146/annurev-ecolsys-110617-062406

[B96] Kniskern J. M. Traw M. B. Bergelson J. (2007). Salicylic acid and jasmonic acid signaling defense pathways reduce natural bacterial diversity on *Arabidopsis thaliana*. *Mol. Plant Microbe Interact.* 20 1512–1522. 10.1094/mpmi-20-12-1512 17990959

[B97] Köberl M. Schmidt R. Ramadan E. M. Bauer R. Berg G. (2013). The microbiome of medicinal plants: diversity and importance for plant growth, quality and health. *Front. Microbiol.* 4:400. 10.3389/fmicb.2013.00400 PMC3868918 24391634

[B98] Koh A. Molinaro A. Stahlman M. Khan M. T. Schmidt C. Manneras-Holm L. (2018). Microbially produced imidazole propionate impairs insulin signaling through mtorc1. *Cell* 175 947.e17–961.e17. 10.1016/j.cell.2018.09.055 30401435

[B99] Kong C. H. Zhang S. Z. Li Y. H. Xia Z. C. Yang X. F. Meiners S. J. (2018). Plant neighbor detection and allelochemical response are driven by root-secreted signaling chemicals. *Nat. Commun.* 9:3867. 10.1038/s41467-018-06429-1 PMC6155373 30250243

[B100] Kong H. G. Song G. C. Sim H.-J. Ryu C.-M. (2020). Achieving similar root microbiota composition in neighbouring plants through airborne signalling. *ISME J.* 15 397–408. 10.1038/s41396-020-00759-z 32973341 PMC8027813

[B101] Koprivova A. Schuck S. Jacoby R. P. Klinkhammer I. Welter B. Leson L. (2019). Root-specific camalexin biosynthesis controls the plant growth-promoting effects of multiple bacterial strains. *Proc. Natl. Acad. Sci. U.S.A.* 116 15735–15744. 10.1073/pnas.1818604116 31311863 PMC6681745

[B102] Korenblum E. Aharoni A. (2019). Phytobiome metabolism: beneficial soil microbes steer crop plants’ secondary metabolism. *Pest. Manag. Sci.* 75 2378–2384. 10.1002/ps.5440 30973666

[B103] Korenblum E. Dong Y. Szymanski J. Panda S. Jozwiak A. Massalha H. (2020). Rhizosphere microbiome mediates systemic root metabolite exudation by root-to-root signaling. *Proc. Natl. Acad. Sci. U.S.A.* 117 3874–3883. 10.1073/pnas.1912130117 32015118 PMC7035606

[B104] Kosmacz M. Sokołowska E. M. Bouzaa S. Skirycz A. (2020). Towards a functional understanding of the plant metabolome. *Curr. Opin. Plant Biol.* 55 47–51. 10.1016/j.pbi.2020.02.005 32224339

[B105] Kostic A. D. Gevers D. Siljander H. Vatanen T. Hyotylainen T. Hamalainen A. M. (2015). The dynamics of the human infant gut microbiome in development and in progression toward type 1 diabetes. *Cell Host Microbe.* 17 260–273. 10.1016/j.chom.2015.01.001 25662751 PMC4689191

[B106] Kozuka T. Sawada Y. Imai H. Kanai M. Hirai M. Y. Mano S. (2020). Regulation of sugar and storage oil metabolism by phytochrome during de-etiolation. *Plant Physiol.* 182 1114–1129. 10.1104/pp.19.00535 31748417 PMC6997681

[B107] Kudjordjie E. N. Sapkota R. Steffensen S. K. Fomsgaard I. S. Nicolaisen M. (2019). Maize synthesized benzoxazinoids affect the host associated microbiome. *Microbiome* 7:59. 10.1186/s40168-019-0677-7 PMC6460791 30975184

[B108] Kurtz Z. D. Muller C. L. Miraldi E. R. Littman D. R. Blaser M. J. Bonneau R. A. (2015). Sparse and compositionally robust inference of microbial ecological networks. *PLoS Comput. Biol.* 11:e1004226. 10.1371/journal.pcbi.1004226 PMC4423992 25950956

[B109] Lamichhane S. Sen P. Dickens A. M. Orešič M. Bertram H. C. (2018). Gut metabolome meets microbiome: a methodological perspective to understand the relationship between host and microbe. *Methods* 149 3–12. 10.1016/j.ymeth.2018.04.029 29715508

[B110] Larsen P. E. Collart F. R. Field D. Meyer F. Keegan K. P. Henry C. S. (2011). Predicted relative metabolomic turnover (prmt): determining metabolic turnover from a coastal marine metagenomic dataset. *Microb. Inform. Exp.* 1:4. 10.1186/2042-5783-1-4 PMC3348665 22587810

[B111] Lavelle A. Sokol H. (2020). Gut microbiota-derived metabolites as key actors in inflammatory bowel disease. *Nat. Rev. Gastroenterol. Hepatol.* 17 223–237. 10.1038/s41575-019-0258-z 32076145

[B112] Lebeis S. L. Paredes S. H. Lundberg D. S. Breakfield N. Gehring J. McDonald M. (2015). Plant microbiome. Salicylic acid modulates colonization of the root microbiome by specific bacterial taxa. *Science* 349 860–864. 10.1126/science.aaa8764 26184915

[B113] Lenzewski N. Mueller P. Meier R. J. Liebsch G. Jensen K. Koop-Jakobsen K. (2018). Dynamics of oxygen and carbon dioxide in rhizospheres of lobelia dortmanna – a planar optode study of belowground gas exchange between plants and sediment. *New Phytol.* 218 131–141. 10.1111/nph.14973 29314005

[B114] Li B. Li Y.-Y. Wu H.-M. Zhang F.-F. Li C.-J. Li X.-X. (2016). Root exudates drive interspecific facilitation by enhancing nodulation and n2 fixation. *Proc. Natl. Acad. Sci. U.S.A.* 113 6496–6501. 10.1073/pnas.1523580113 27217575 PMC4988560

[B115] Liang D. Li M. Wei R. Wang J. Li Y. Jia W. (2019). Strategy for intercorrelation identification between metabolome and microbiome. *Anal. Chem.* 91 14424–14432. 10.1021/acs.analchem.9b02948 31638380

[B116] Liu H. Brettell L. E. (2019). Plant defense by voc-induced microbial priming. *Trends Plant Sci.* 24 187–189. 10.1016/j.tplants.2019.01.008 30738790

[B117] Liu H. Brettell L. E. Qiu Z. Singh B. K. (2020). Microbiome-mediated stress resistance in plants. *Trends Plant Sci.* 25 733–743. 10.1016/j.tplants.2020.03.014 32345569

[B118] Liu H. Carvalhais L. C. Schenk P. M. Dennis P. G. (2017). Effects of jasmonic acid signalling on the wheat microbiome differ between body sites. *Sci. Rep.* 7:41766. 10.1038/srep41766 PMC5278374 28134326

[B119] Liu Y. X. Qin Y. Bai Y. (2019). Reductionist synthetic community approaches in root microbiome research. *Curr. Opin. Microbiol.* 49 97–102. 10.1016/j.mib.2019.10.010 31734603

[B120] Liu Y. X. Qin Y. Chen T. Lu M. Qian X. Guo X. (2020). A practical guide to amplicon and metagenomic analysis of microbiome data. *Protein Cell* 10.1007/s13238-020-00724-8 Online ahead of print PMC8106563 32394199

[B121] Lock E. F. Hoadley K. A. Marron J. S. Nobel A. B. (2013). Joint and individual variation explained (jive) for integrated analysis of multiple data types. *Ann. Appl. Stat.* 7 523–542. 10.1214/12-AOAS597 23745156 PMC3671601

[B122] Lovell D. Pawlowsky-Glahn V. Egozcue J. J. Marguerat S. Bahler J. (2015). Proportionality: a valid alternative to correlation for relative data. *PLoS Comput. Biol.* 11:e1004075. 10.1371/journal.pcbi.1004075 PMC4361748 25775355

[B123] Lu W. Su X. Klein M. S. Lewis I. A. Fiehn O. Rabinowitz J. D. (2017). Metabolite measurement: pitfalls to avoid and practices to follow. *Annu. Rev. Biochem.* 86 277–304. 10.1146/annurev-biochem-061516-044952 28654323 PMC5734093

[B124] Lucaciu R. Pelikan C. Gerner S. M. Zioutis C. Köstlbacher S. Marx H. (2019). A bioinformatics guide to plant microbiome analysis. *Front. Plant Sci.* 10:1313. 10.3389/fpls.2019.01313 PMC6819368 31708944

[B125] Lundberg D. S. Teixeira P. (2018). Root-exuded coumarin shapes the root microbiome. *Proc. Natl. Acad. Sci. U.S.A.* 115 5629–5631. 10.1073/pnas.1805944115 29764997 PMC5984549

[B126] Luo P. Yin P. Zhang W. Zhou L. Lu X. Lin X. (2016). Optimization of large-scale pseudotargeted metabolomics method based on liquid chromatography–mass spectrometry. *J. Chromatogr. A* 1437 127–136. 10.1016/j.chroma.2016.01.078 26877181

[B127] Maggini V. Bandeira Reidel R. V. De Leo M. Mengoni A. Gallo E. R. Miceli E. (2019a). Volatile profile of echinacea purpurea plants after in vitro endophyte infection. *Nat. Prod. Res.* 34 2232–2237. 10.1080/14786419.2019.1579810 30908079

[B128] Maggini V. De Leo M. Granchi C. Tuccinardi T. Mengoni A. Gallo E. R. (2019b). The influence of echinacea purpurea leaf microbiota on chicoric acid level. *Sci. Rep.* 9:10897. 10.1038/s41598-019-47329-8 PMC6659708 31350520

[B129] Maggini V. De Leo M. Mengoni A. Gallo E. R. Miceli E. Reidel R. V. B. (2017). Plant-endophytes interaction influences the secondary metabolism in echinacea purpurea (l.) moench: an in vitro model. *Sci. Rep.* 7:16924. 10.1038/s41598-017-17110-w PMC5717142 29208923

[B130] Maggini V. Mengoni A. Bogani P. Firenzuoli F. Fani R. (2020). Promoting model systems of microbiota-medicinal plant interactions. *Trends Plant Sci.* 25 223–225. 10.1016/j.tplants.2019.12.013 31948792

[B131] Maggini V. Miceli E. Fagorzi C. Maida I. Fondi M. Perrin E. (2018). Antagonism and antibiotic resistance drive a species-specific plant microbiota differentiation in echinacea spp. *FEMS Microbiol. Ecol.* 94 1–18. 10.1093/femsec/fiy118 29912319

[B132] Magnúsdóttir S. Heinken A. Kutt L. Ravcheev D. A. Bauer E. Noronha A. (2017). Generation of genome-scale metabolic reconstructions for 773 members of the human gut microbiota. *Nat. Biotechnol.* 35 81–89. 10.1038/nbt.3703 27893703

[B133] Mallick H. Franzosa E. A. McLver L. J. Banerjee S. Sirota-Madi A. Kostic A. D. (2019). Predictive metabolomic profiling of microbial communities using amplicon or metagenomic sequences. *Nat. Commun.* 10:3136. 10.1038/s41467-019-10927-1 PMC6637180 31316056

[B134] Mao S.-Y. Huo W.-J. Zhu W.-Y. (2016). Microbiome–metabolome analysis reveals unhealthy alterations in the composition and metabolism of ruminal microbiota with increasing dietary grain in a goat model. *Environ. Microbiol.* 18 525–541. 10.1111/1462-2920.12724 25471302

[B135] Mariano D. C. Pereira F. L. Aguiar E. L. Oliveira L. C. Benevides L. Guimaraes L. C. (2016). Simba: a web tool for managing bacterial genome assembly generated by ion pgm sequencing technology. *BMC Bioinformatics* 17:456. 10.1186/s12859-016-1344-7 PMC5249034 28105921

[B136] Massalha H. Korenblum E. Malitsky S. Shapiro O. H. Aharoni A. (2017). Live imaging of root-bacteria interactions in a microfluidics setup. *Proc. Natl. Acad. Sci. U.S.A.* 114 4549–4554. 10.1073/pnas.1618584114 28348235 PMC5410799

[B137] Mastan A. Bharadwaj R. K. B. Kushwaha R. K. Vivek Babu C. S. (2019). Functional fungal endophytes in coleus forskohlii regulate labdane diterpene biosynthesis for elevated forskolin accumulation in roots. *Microb. Ecol.* 78 914–926. 10.1007/s00248-019-01376-w 31001657

[B138] Masteling R. Lombard L. de Boer W. Raaijmakers J. M. Dini-Andreote F. (2019). Harnessing the microbiome to control plant parasitic weeds. *Curr. Opin. Microbiol.* 49 26–33. 10.1016/j.mib.2019.09.006 31654911 PMC6906922

[B139] Masuda K. Abouleila Y. Ali A. Yanagida T. Masujima T. (2018). “Live single-cell mass spectrometry (lsc-ms) for plant metabolomics,” in *Plant Metabolomics: Methods and Protocols*, eds António C. (New York, NY: Springer New York). 10.1007/978-1-4939-7819-9_19 29761445

[B140] McGregor K. Labbe A. Greenwood C. M. T. (2020). Mdine: a model to estimate differential co-occurrence networks in microbiome studies. *Bioinformatics* 36 1840–1847. 10.1093/bioinformatics/btz824 31697315 PMC7075537

[B141] McHardy I. H. Goudarzi M. Tong M. Ruegger P. M. Schwager E. Weger J. R. (2013). Integrative analysis of the microbiome and metabolome of the human intestinal mucosal surface reveals exquisite inter-relationships. *Microbiome* 1:17. 10.1186/2049-2618-1-17 PMC3971612 24450808

[B142] McIver L. J. Abu-Ali G. Franzosa E. A. Schwager R. Morgan X. C. Waldron L. (2018). Biobakery: a meta’omic analysis environment. *Bioinformatics* 34 1235–1237. 10.1093/bioinformatics/btx754 29194469 PMC6030947

[B143] Mehmood A. Hussain A. Irshad M. Hamayun M. Iqbal A. Tawab A. (2020). Yucasin and cinnamic acid inhibit iaa and flavonoids biosynthesis minimizing interaction between maize and endophyteaspergillus nomius. *Symbiosis* 12 149–160. 10.1007/s13199-020-00690-z

[B144] Melnik A. V. da Silva R. R. Hyde E. R. Aksenov A. A. Vargas F. Bouslimani A. (2017). Coupling targeted and untargeted mass spectrometry for metabolome-microbiome-wide association studies of human fecal samples. *Anal. Chem.* 89 7549–7559. 10.1021/acs.analchem.7b01381 28628333

[B145] Mendes-Soares H. Mundy M. Soares L. M. Chia N. (2016). Mminte: an application for predicting metabolic interactions among the microbial species in a community. *BMC Bioinformatics* 17:343. 10.1186/s12859-016-1230-3 PMC5009493 27590448

[B146] Millet L. J. Aufrecht J. Labbe J. Uehling J. Vilgalys R. Estes M. L. (2019). Increasing access to microfluidics for studying fungi and other branched biological structures. *Fungal Biol. Biotechnol.* 6:1. 10.1186/s40694-019-0071-z PMC6556955 31198578

[B147] Misra B. B. (2020). The connection and disconnection between microbiome and metabolome: a critical appraisal in clinical research. *Biol. Res. Nurs.* 22 561–576. 10.1177/1099800420903083 32013533

[B148] Morton J. T. Aksenov A. A. Nothias L. F. Foulds J. R. Quinn R. A. Badri M. H. (2019). Learning representations of microbe-metabolite interactions. *Nat. Methods* 16 1306–1314. 10.1038/s41592-019-0616-3 31686038 PMC6884698

[B149] Nascimento F. X. Rossi M. J. Glick B. R. (2018). Ethylene and 1-aminocyclopropane-1-carboxylate (acc) in plant–bacterial interactions. *Front. Plant Sci.* 9:114. 10.3389/fpls.2018.00114 PMC5827301 29520283

[B150] Nasir F. Li W. Tran L.-S. P. Tian C. (2020). Does karrikin signaling shape the rhizomicrobiome via the strigolactone biosynthetic pathway? *Trends Plant Sci.* 25 1184–1187. 10.1016/j.tplants.2020.08.005 32888808

[B151] Nasir F. Shi S. Tian L. Chang C. Ma L. Li X. (2019). Strigolactones shape the rhizomicrobiome in rice (*Oryza sativa*). *Plant Sci.* 286 118–133. 10.1016/j.plantsci.2019.05.016 31300137

[B152] Naylor D. Coleman-Derr D. (2017). Drought stress and root-associated bacterial communities. *Front. Plant Sci.* 8:2223. 10.3389/fpls.2017.02223 PMC5767233 29375600

[B153] Nemet I. Saha P. P. Gupta N. Zhu W. Romano K. A. Skye S. M. (2020). A cardiovascular disease-linked gut microbial metabolite acts via adrenergic receptors. *Cell* 180 862.e22–877.e22. 10.1016/j.cell.2020.02.016 32142679 PMC7402401

[B154] Ni Y. Yu G. Chen H. Deng Y. Wells P. M. Steves C. J. (2020). M2ia: a web server for microbiome and metabolome integrative analysis. *Bioinformatics*. 36 3493–3498. 10.1093/bioinformatics/btaa188 32176258

[B155] Nilsson R. H. Anslan S. Bahram M. Wurzbacher C. Baldrian P. Tedersoo L. (2019). Mycobiome diversity: high-throughput sequencing and identification of fungi. *Nat. Rev. Microbiol.* 17 95–109. 10.1038/s41579-018-0116-y 30442909

[B156] Noecker C. Chiu H.-C. McNally C. P. Borenstein E. (2019). Defining and evaluating microbial contributions to metabolite variation in microbiome-metabolome association studies. *mSystems* 4:e579-19. 10.1128/mSystems.00579-19 PMC6918031 31848305

[B157] Noecker C. Eng A. Srinivasan S. Theriot C. M. Young V. B. Jansson J. K. (2016). Metabolic model-based integration of microbiome taxonomic and metabolomic profiles elucidates mechanistic links between ecological and metabolic variation. *mSystems* 1:e00013-15. 10.1128/mSystems.00013-15 PMC4883586 27239563

[B158] Noirot-Gros M. F. Shinde S. V. Akins C. Johnson J. L. Zerbs S. Wilton R. (2020). Functional imaging of microbial interactions with tree roots using a microfluidics setup. *Front. Plant Sci.* 11:408. 10.3389/fpls.2020.00408 PMC7174594 32351525

[B159] Okutani F. Hamamoto S. Aoki Y. Nakayasu M. Nihei N. Nishimura T. (2020). Rhizosphere modelling reveals spatiotemporal distribution of daidzein shaping soybean rhizosphere bacterial community. *Plant Cell Environ.* 43 1036–1046. 10.1111/pce.13708 31875335

[B160] Ortíz-Castro R. Contreras-Cornejo H. A. Macías-Rodríguez L. López-Bucio J. (2009). The role of microbial signals in plant growth and development. *Plant Signal. Behav.* 4 701–712. 10.4161/psb.4.8.9047 19820333 PMC2801380

[B161] Pagnani G. Pellegrini M. Galieni A. D’Egidio S. Matteucci F. Ricci A. (2018). Plant growth-promoting rhizobacteria (pgpr) in cannabis sativa ‘finola’ cultivation: an alternative fertilization strategy to improve plant growth and quality characteristics. *Indust. Crops Prod.* 123 75–83. 10.1016/j.indcrop.2018.06.033

[B162] Pangesti N. Reichelt M. van de Mortel J. E. Kapsomenou E. Gershenzon J. van Loon J. J. (2016). Jasmonic acid and ethylene signaling pathways regulate glucosinolate levels in plants during rhizobacteria-induced systemic resistance against a leaf-chewing herbivore. *J. Chem. Ecol.* 42 1212–1225. 10.1007/s10886-016-0787-7 27848154 PMC5148788

[B163] Pascale A. Proietti S. Pantelides I. S. Stringlis I. A. (2020). Modulation of the root microbiome by plant molecules: the basis for targeted disease suppression and plant growth promotion. *Front. Plant Sci.* 10:1741. 10.3389/fpls.2019.01741 PMC6992662 32038698

[B164] Pei Y. C. Siemann E. Tian B. L. Ding J. Q. (2020). Root flavonoids are related to enhanced amf colonization of an invasive tree. *AoB Plants* 12:laa002. 10.1093/aobpla/plaa002 PMC7015461 32071712

[B165] Peñuelas J. Farré-Armengol G. Llusia J. Gargallo-Garriga A. Rico L. Sardans J. (2014). Removal of floral microbiota reduces floral terpene emissions. *Sci. Rep.* 4:6727. 10.1038/srep06727 PMC4205883 25335793

[B166] Perez-Jaramillo J. E. de Hollander M. Ramirez C. A. Mendes R. Raaijmakers J. M. Carrion V. J. (2019). Deciphering rhizosphere microbiome assembly of wild and modern common bean (*Phaseolus vulgaris*) in native and agricultural soils from colombia. *Microbiome* 7:114. 10.1186/s40168-019-0727-1 PMC6694607 31412927

[B167] Peter A. Pola S. P. Sandeep B. Rao B. (2019). *Antimicrobial and Anti-Quorum Sensing Activities of Medicinal Plants.* Singapore: Springer.

[B168] Petriacq P. Williams A. Cotton A. McFarlane A. E. Rolfe S. A. Ton J. (2017). Metabolite profiling of non-sterile rhizosphere soil. *Plant J.* 92 147–162. 10.1111/tpj.13639 28742258 PMC5639361

[B169] Piasecka A. Jedrzejczak-Rey N. Bednarek P. (2015). Secondary metabolites in plant innate immunity: conserved function of divergent chemicals. *New Phytol.* 206 948–964. 10.1111/nph.13325 25659829

[B170] Pini F. East A. K. Appia-Ayme C. Tomek J. Karunakaran R. Mendoza-Suarez M. (2017). Bacterial biosensors for in vivo spatiotemporal mapping of root secretion. *Plant Physiol.* 174 1289–1306. 10.1104/pp.16.01302 28495892 PMC5490882

[B171] Rajniak J. Giehl R. F. H. Chang E. Murgia I. von Wiren N. Sattely E. S. (2018). Biosynthesis of redox-active metabolites in response to iron deficiency in plants. *Nat. Chem. Biol.* 14 442–450. 10.1038/s41589-018-0019-2 29581584 PMC6693505

[B172] Ray S. Singh J. Rajput R. S. Yadav S. Singh S. Singh H. B. (2019). “A thorough comprehension of host endophytic interaction entailing the biospherical benefits: a metabolomic perspective,” in *Endophytes and Secondary Metabolites*, ed. Jha S. (Cham: Springer International Publishing).

[B173] Ray T. Pandey S. S. Pandey A. Srivastava M. Shanker K. Kalra A. (2019). Endophytic consortium with diverse gene-regulating capabilities of benzylisoquinoline alkaloids biosynthetic pathway can enhance endogenous morphine biosynthesis in papaver somniferum. *Front. Microbiol.* 10:925. 10.3389/fmicb.2019.00925 PMC6503101 31114562

[B174] Raza W. Wang J. Jousset A. Friman V.-P. Mei X. Wang S. (2020). Bacterial community richness shifts the balance between volatile organic compound-mediated microbe–pathogen and microbe–plant interactions. *Proc. R. Soc. B Biol. Sci.* 287:20200403. 10.1098/rspb.2020.0403 PMC7211448 32290797

[B175] Reshef D. N. Reshef Y. A. Finucane H. K. Grossman S. R. McVean G. Turnbaugh P. J. (2011). Detecting novel associations in large data sets. *Science* 334 1518–1524. 10.1126/science.1205438 22174245 PMC3325791

[B176] Rodriguez P. A. Rothballer M. Chowdhury S. P. Nussbaumer T. Gutjahr C. Falter-Braun P. (2019). Systems biology of plant-microbiome interactions. *Mol. Plant* 12 804–821. 10.1016/j.molp.2019.05.006 31128275

[B177] Rognes T. Flouri T. Nichols B. Quince C. Mahe F. (2016). Vsearch: a versatile open source tool for metagenomics. *PeerJ.* 4:e2584. 10.7717/peerj.2584 PMC5075697 27781170

[B178] Sasse J. Martinoia E. Northen T. (2018). Feed your friends: do plant exudates shape the root microbiome? *Trends Plant Sci.* 23 25–41. 10.1016/j.tplants.2017.09.003 29050989

[B179] Schandry N. Becker C. (2020). Allelopathic plants: models for studying plant–interkingdom interactions. *Trends Plant Sci.* 25 176–185. 10.1016/j.tplants.2019.11.004 31837955

[B180] Schenkel D. Deveau A. Niimi J. Mariotte P. Vitra A. Meisser M. (2019). Linking soil’s volatilome to microbes and plant roots highlights the importance of microbes as emitters of belowground volatile signals. *Environ. Microbiol.* 10.1111/1462-2920.14599 Online ahead of print 30895716

[B181] Schlechter R. O. Miebach M. Remus-Emsermann M. N. P. (2019). Driving factors of epiphytic bacterial communities: a review. *J. Adv. Res.* 19 57–65. 10.1016/j.jare.2019.03.003 31341670 PMC6630024

[B182] Schulz-Bohm K. Gerards S. Hundscheid M. Melenhorst J. de Boer W. Garbeva P. (2018). Calling from distance: attraction of soil bacteria by plant root volatiles. *ISME J.* 12 1252–1262. 10.1038/s41396-017-0035-3 29358736 PMC5931972

[B183] Schütz V. Bigler L. Girel S. Laschke L. Sicker D. Schulz M. (2019). Conversions of benzoxazinoids and downstream metabolites by soil microorganisms. *Front. Ecol. Evol.* 7:238. 10.3389/fevo.2019.00238

[B184] Shtark O. Y. Shishova M. F. Povydysh M. N. Avdeeva G. S. Zhukov V. A. Tikhonovich I. A. (2018). Strigolactones as regulators of symbiotrophy of plants and microorganisms. *Russ. J. Plant Physiol.* 65 151–167. 10.1134/S1021443718020073

[B185] Smilde A. K. Måge I. Næs T. Hankemeier T. Lips M. A. Kiers H. A. L. (2017). Common and distinct components in data fusion. *J. Chemom.* 31:e2900. 10.1002/cem.2900

[B186] Sokol N. W. Kuebbing S. E. Karlsen-Ayala E. Bradford M. A. (2019). Evidence for the primacy of living root inputs, not root or shoot litter, in forming soil organic carbon. *New Phytol.* 221 233–246. 10.1111/nph.15361 30067293

[B187] Stassen M. J. J. Hsu S.-H. Pieterse C. M. J. Stringlis I. A. (2020). Coumarin communication along the microbiome–root–shoot axis. *Trends Plant Sci.* 26 169–183. 10.1016/j.tplants.2020.09.008 33023832

[B188] Stefanowicz A. M. Stanek M. Nobis M. Zubek S. (2017). Few effects of invasive plants reynoutria japonica, rudbeckia laciniata and solidago gigantea on soil physical and chemical properties. *Sci. Total Environ.* 574 938–946. 10.1016/j.scitotenv.2016.09.120 27665453

[B189] Stringlis I. A. de Jonge R. Pieterse C. M. J. (2019). The age of coumarins in plant-microbe interactions. *Plant Cell Physiol.* 60 1405–1419. 10.1093/pcp/pcz076 31076771 PMC6915228

[B190] Stringlis I. A. Yu K. Feussner K. de Jonge R. Van Bentum S. Van Verk M. C. (2018). Myb72-dependent coumarin exudation shapes root microbiome assembly to promote plant health. *Proc. Natl. Acad. Sci. U.S.A.* 115 E5213–E5222. 10.1073/pnas.1722335115 29686086 PMC5984513

[B191] Sugiyama A. (2019). The soybean rhizosphere: metabolites, microbes, and beyond-a review. *J. Adv. Res.* 19 67–73. 10.1016/j.jare.2019.03.005 31341671 PMC6630087

[B192] Sugiyama A. Yazaki K. (2014). Flavonoids in plant rhizospheres: secretion, fate and their effects on biological communication. *Plant Biotechnol.* 31 431–443. 10.5511/plantbiotechnology.14.0917a

[B193] Szoboszlay M. White-Monsant A. Moe L. A. (2016). The effect of root exudate 7,4’-dihydroxyflavone and naringenin on soil bacterial community structure. *PLoS One* 11:e0146555. 10.1371/journal.pone.0146555 PMC4709137 26752410

[B194] Taghinasab M. Jabaji S. (2020). Cannabis microbiome and the role of endophytes in modulating the production of secondary metabolites: an overview. *Microorganisms* 8:355. 10.3390/microorganisms8030355 PMC7143057 32131457

[B195] Tahir A. T. Fatmi Q. Nosheen A. Imtiaz M. Khan S (2019). “Metabolomic approaches in plant research,” in *Essentials of Bioinformatics, Volume III: In Silico Life Sciences: Agriculture*, eds Hakeem K. R. Shaik N. A. Banaganapalli B. (Cham: Springer International Publishing).

[B196] Tian T. Reverdy A. She Q. Sun B. Chai Y. (2020). The role of rhizodeposits in shaping rhizomicrobiome. *Environ. Microbiol. Rep.* 12 160–172. 10.1111/1758-2229.12816 31858707

[B197] Tian Y. Amand S. Buisson D. Kunz C. Hachette F. Dupont J. (2014). The fungal leaf endophyte paraconiothyrium variabile specifically metabolizes the host-plant metabolome for its own benefit. *Phytochemistry* 108 95–101. 10.1016/j.phytochem.2014.09.021 25446235

[B198] Tidke S. A. Kiran S. Giridhar P. Gokare R. A. (2019). “Current understanding and future perspectives of endophytic microbes vis-a-vis production of secondary metabolites,” in *Endophytes and Secondary Metabolites*, ed. Jha S. (Cham: Springer International Publishing).

[B199] Trda L. Janda M. Mackova D. Pospichalova R. Dobrev P. I. Burketova L. (2019). Dual mode of the saponin aescin in plant protection: antifungal agent and plant defense elicitor. *Front. Plant Sci.* 10:1448. 10.3389/fpls.2019.01448 PMC6893899 31850004

[B200] Ullrich C. I. Aloni R. Saeed M. E. M. Ullrich W. Efferth T. (2019). Comparison between tumors in plants and human beings: mechanisms of tumor development and therapy with secondary plant metabolites. *Phytomedicine* 64:153081. 10.1016/j.phymed.2019.153081 31568956

[B201] van der Kloet F. M. Sebastian-Leon P. Conesa A. Smilde A. K. Westerhuis J. A. (2016). Separating common from distinctive variation. *BMC Bioinformatics* 17 (Suppl. 5):195. 10.1186/s12859-016-1037-2 PMC4905617 27294690

[B202] Van Deynze A. Zamora P. Delaux P. M. Heitmann C. Jayaraman D. Rajasekar S. (2018). Nitrogen fixation in a landrace of maize is supported by a mucilage-associated diazotrophic microbiota. *PLoS Biol.* 16:e2006352. 10.1371/journal.pbio.2006352 PMC6080747 30086128

[B203] Varoquaux N. Cole B. Gao C. Pierroz G. Baker C. R. Patel D. (2019). Transcriptomic analysis of field-droughted sorghum from seedling to maturity reveals biotic and metabolic responses. *Proc Natl Acad Sci U S A.* 10.1073/pnas.1907500116 Online ahead of print PMC6936495 31806758

[B204] Venturi V. Keel C. (2016). Signaling in the rhizosphere. *Trends Plant Sci.* 21 187–198. 10.1016/j.tplants.2016.01.005 26832945

[B205] Verbeek J. D. Kotanen P. M. (2019). Soil-mediated impacts of an invasive thistle inhibit the recruitment of certain native plants. *Oecologia* 190 619–628. 10.1007/s00442-019-04435-8 31197481

[B206] Vives-Peris V. de Ollas C. Gomez-Cadenas A. Perez-Clemente R. M. (2020). Root exudates: from plant to rhizosphere and beyond. *Plant Cell Rep.* 39 3–17. 10.1007/s00299-019-02447-5 31346716

[B207] Voges M. Bai Y. Schulze-Lefert P. Sattely E. S. (2019). Plant-derived coumarins shape the composition of an *Arabidopsis* synthetic root microbiome. *Proc. Natl. Acad. Sci. U.S.A.* 116 12558–12565. 10.1073/pnas.1820691116 31152139 PMC6589675

[B208] Wang D. Xu Z. Zhang G. Xia L. Dong X. Li Q. (2019). A genomic island in a plant beneficial rhizobacterium encodes novel antimicrobial fatty acids and a self-protection shield to enhance its competition. *Environ. Microbiol.* 10.1111/1462-2920.14683 Online ahead of print 31106958

[B209] Wang P. Niu B. (2019). Plant specialized metabolites modulate root microbiomes. *Sci. China Life Sci.* 62 1111–1113. 10.1007/s11427-019-9579-6 31290099

[B210] Wang W. Yang J. Zhang J. Liu Y. X. Tian C. Qu B. (2020). An *Arabidopsis* secondary metabolite directly targets expression of the bacterial type iii secretion system to inhibit bacterial virulence. *Cell Host Microbe* 27 601.e7–613.e7. 10.1016/j.chom.2020.03.004 32272078

[B211] Wasternack C. Hause B. (2013). Jasmonates: biosynthesis, perception, signal transduction and action in plant stress response, growth and development. An update to the 2007 review in annals of botany. *Ann. Bot.* 111 1021–1058. 10.1093/aob/mct067 23558912 PMC3662512

[B212] Weiss S. Van Treuren W. Lozupone C. Faust K. Friedman J. Deng Y. (2016). Correlation detection strategies in microbial data sets vary widely in sensitivity and precision. *ISME J.* 10 1669–1681. 10.1038/ismej.2015.235 26905627 PMC4918442

[B213] White L. J. Ge X. Brözel V. S. Subramanian S. (2017). Root isoflavonoids and hairy root transformation influence key bacterial taxa in the soybean rhizosphere. *Environ. Microbiol.* 19 1391–1406. 10.1111/1462-2920.13602 27871141

[B214] Williams A. de Vries F. T. (2020). Plant root exudation under drought: implications for ecosystem functioning. *New Phytol.* 225 1899–1905. 10.1111/nph.16223 31571220

[B215] Xu L. Naylor D. Dong Z. Simmon T. Pierroz G. Hixson K. K. (2018). Drought delays development of the sorghum root microbiome and enriches for monoderm bacteria. *Proc. Natl. Acad. Sci. U.S.A.* 115:E4952. 10.1073/pnas.1807275115 PMC5939072 29666229

[B216] Yang L. Wen K. S. Ruan X. Zhao Y. X. Wei F. Wang Q. (2018). Response of plant secondary metabolites to environmental factors. *Molecules* 23:762. 10.3390/molecules23040762 PMC6017249 29584636

[B217] Yang W. Zhang D. Cai X. Xia L. Luo Y. Cheng X. (2019). Significant alterations in soil fungal communities along a chronosequence of spartina alterniflora invasion in a chinese yellow sea coastal wetland. *Sci. Total Environ.* 693:133548. 10.1016/j.scitotenv.2019.07.354 31369894

[B218] You M. Fang S. M. MacDonald J. Xu J. Yuan Z. C. (2020). Isolation and characterization of Burkholderia cenocepacia CR318, a phosphate solubilizing bacterium promoting corn growth. *Microbiol. Res.* 233:126395. 10.1016/j.micres.2019.126395 31865096

[B219] Youens-Clark K. Bomhoff M. Ponsero A. J. Wood-Charlson E. M. Lynch J. Choi I. (2019). Imicrobe: tools and data-dreaiven discovery platform for the microbiome sciences. *Gigascience* 8:giz083. 10.1093/gigascience/giz083 PMC6615980 31289831

[B220] Yuan J. Zhao J. Wen T. Zhao M. Li R. Goossens P. (2018). Root exudates drive the soil-borne legacy of aboveground pathogen infection. *Microbiome* 6:156. 10.1186/s40168-018-0537-x PMC6136170 30208962

[B221] Zhalnina K. Louie K. B. Hao Z. Mansoori N. da Rocha U. N. Shi S. (2018). Dynamic root exudate chemistry and microbial substrate preferences drive patterns in rhizosphere microbial community assembly. *Nat. Microbiol.* 3 470–480. 10.1038/s41564-018-0129-3 29556109

[B222] Zhan Z. J. Tian T. Xu Y. L. Yu H. F. Zhang C. X. Zhang Z. D. (2019). Biotransformation of huperzine b by a fungal endophyte of huperzia serrata. *Chem. Biodivers.* 16:e1900299. 10.1002/cbdv.201900299 31287220

[B223] Zhang B. Zhang J. Liu Y. Shi P. Wei G. (2018). Co-occurrence patterns of soybean rhizosphere microbiome at a continental scale. *Soil Biol. Biochem.* 118 178–186. 10.1016/j.soilbio.2017.12.011

[B224] Zhang H. Kim M. S. Krishnamachari V. Payton P. Sun Y. Crimson M. (2007). Rhizobacterial volatile emissions regulate auxin homeostasis and cell expansion in *Arabidopsis*. *Planta* 226 839–851. 10.1007/s00425-007-0530-2 17497164

[B225] Zhang J. Liu Y. X. Zhang N. Hu B. Jin T. Xu H. (2019). Nrt1.1b is associated with root microbiota composition and nitrogen use in field-grown rice. *Nat. Biotechnol.* 37 676–684. 10.1038/s41587-019-0104-4 31036930

[B226] Zhang Y. Li S. S. Li H. X. Wang R. R. Zhang K. Q. Xu J. (2020). Fungal-nematode interactions: diversity, ecology and biocontrol prospects in agriculture. *J. Fungi.* 6:206. 10.3390/jof6040206 PMC7711821 33020457

[B227] Zhang Y. Xu J. Riera N. Jin T. Li J. Wang N. (2017). Huanglongbing impairs the rhizosphere-to-rhizoplane enrichment process of the citrus root-associated microbiome. *Microbiome* 5:97. 10.1186/s40168-017-0304-4 PMC5553657 28797279

[B228] Zheng Q. Bartow-McKenney C. Meisel J. S. Grice E. A. (2018). Hmmufotu: an hmm and phylogenetic placement based ultra-fast taxonomic assignment and otu picking tool for microbiome amplicon sequencing studies. *Genome Biol.* 19:82. 10.1186/s13059-018-1450-0 PMC6020470 29950165

[B229] Zhou F. Emonet A. Dénervaud Tendon V. Marhavy P. Wu D. Lahaye T. (2020). Co-incidence of damage and microbial patterns controls localized immune responses in roots. *Cell* 180 440.e18–453.e18. 10.1016/j.cell.2020.01.013 32032516 PMC7042715

[B230] Zhou F. Pichersky E. (2020). More is better: the diversity of terpene metabolism in plants. *Curr. Opin. Plant Biol.* 55 1–10. 10.1016/j.pbi.2020.01.005 32088555

[B231] Zhou J.-L. Xu J. Jiao A.-G. Yang L. Chen J. Callac P. (2019). Patterns of PCR amplification artifacts of the fungal barcode marker in a hybrid mushroom. *Front. Microbiol.* 10:2686. 10.3389/fmicb.2019.02686 PMC6877668 31803173

[B232] Zhou J. Y. Sun K. Chen F. Yuan J. Li X. Dai C. C. (2018). Endophytic *Pseudomonas* induces metabolic flux changes that enhance medicinal sesquiterpenoid accumulation in atractylodes lancea. *Plant Physiol. Biochem.* 130 473–481. 10.1016/j.plaphy.2018.07.016 30081324

